# ﻿Molecular phylogeny and morphology reveal four novel species in Cordycipitaceae in China

**DOI:** 10.3897/mycokeys.116.147006

**Published:** 2025-04-09

**Authors:** Jing Bu, De-Ping Wei, Zheng-Hui Liu, Yang Yang, Zhong-Liang Liu, Ji-Chuan Kang, Xing-Can Peng, Shi-Wen Xie, He-Gui Zhang, Zhang-Jiang He, Shi-Ke Huang, Xian Zhang, Kevin D. Hyde, Nalin N. Wijayawardene, Ting-Chi Wen

**Affiliations:** 1 School of Pharmacy, Guizhou University, Guiyang 550025, Guizhou Province, China; 2 State Key Laboratory of Green Pesticide, Key Laboratory of Green Pesticide and Agricultural Bioengineering, Ministry of Education, Guizhou University, Guiyang 550025, Guizhou Province, China; 3 Engineering Research Center of Southwest Bio-Pharmaceutical Resources, Ministry of Education, Guizhou University, Guiyang 550025, Guizhou Province, China; 4 Guizhou Guiwang Biotechnology Co., Ltd, Daozhen 563599, Guizhou Province, China; 5 Center of Excellence in Fungal Research, Mae Fah Luang University, Chiang Rai 57100, Thailand; 6 School of Science, Mae Fah Luang University, Chiang Rai 57100, Thailand; 7 College of Resources and Environment, Zunyi Normal University, Zunyi 563000, Guizhou Province, China; 8 Center for Yunnan Plateau Biological Resources Protection and Uilization, College of Biology and Food Engineering, Qujing Normal University, Qujing, 655011, Yunnan Province, China

**Keywords:** Entomopathogenic fungi, four new species, morphology, phylogeny

## Abstract

Cordycipitaceae is a well-known family in Hypocreales, comprising numerous arthropod-pathogenic species. Many taxa in this family have been identified and described through integrated morphological and molecular analyses. In this study, phylogenetic analyses using nrLSU, ITS, nrSSU, 3P*_TEF*, *rpb1*, and *rpb2* revealed a new species, *Pleurodesmosporasanduensis*, and a new collection of *Akanthomycesbaishanensis*. Additionally, a concatenated 5P_*TEF*+3P*_TEF*+*rpb1*+*MCM7* dataset was employed to clarify interspecific relationships within *Samsoniella*, identifying three new species: *Samsoniellalurida*, *S.subasiatica*, and *S.torquatistipitata*. Detailed morphological descriptions and illustrations are provided for each studied species.

## ﻿Introduction

Cordycipitaceae belongs to Hypocreales (Hypocreomycetidae, Sordariomycetes), and currently it includes 38 genera. Their phylogenetic relationships have been confirmed through molecular and morphological studies (Sung et al. 2001, 2007; Zare and Gams 2016; Kepler et al. 2017; Zhang et al. 2017, 2021; Mongkolsamrit et al. 2018, 2020, 2021, 2022, 2023; Flakus et al. 2019; Wei et al. 2019; Thanakitpipattana et al. 2020, 2022; Wang et al. 2020; Chen et al. 2021a, 2025; Alves et al. 2022; Araújo et al. 2022; Crous et al. 2023a; Guerra-Mateo et al. 2023; Kobmoo et al. 2023; Custódio and Pereira 2024; Hyde et al. 2024; Khonsanit et al. 2024). Most Cordycipitaceae species are known as pathogens of insects and spiders, while others are reported as hyperparasites on fungi and lichens or are isolated from soil, dung, air, and plant materials (Kepler et al. 2017; Wang et al. 2020; Wei et al. 2022). To adapt to the diverse hosts and habitats, members of Cordycipitaceae have evolved with a wide variety of teleomorphic and anamorphic characteristics (e.g., *Akanthomyces*, *Samsoniella*, and *Pleurodesmospora*).

The genus *Akanthomyces* was introduced by Lebert (1858), typifying with *A.aculeatus* (Mains 1950), and currently 60 epithets are listed in Index Fungorum (http://www.indexfungorum.org/, retrieval on 18 March 2025). Species of *Akanthomyces* are characterised by forming superficial, yellow perithecia on mycelial mat covering spider hosts and the filiform, intact ascospores (Boudier 1885; Mongkolsamrit et al. 2018). Later, the morphological diversity of *Akanthomyces* was broadened to include species with isaria-like and lecanicillium-like anamorphs based on phylogenetic evidence (Mongkolsamrit et al. 2018; Vinit et al. 2018; Chen et al. 2020a, b, 2022). The members of the genus have been reported as insect parasites, plant pathogens, fungicolous organisms, and inhabitants of peat, water, and rust (Wang et al. 2024b). Khonsanit et al. (2024) introduced four genera (i.e., *Arachnidicola*, *Lecanicillium*, *Akanthomyces*, and *Kanoksria*) to accommodate *Akanthomyces* species that are not congeneric with *Akanthomycessensu stricto*.

*Samsoniella* was established by Mongkolsamrit et al. (2018) to accommodate *S.alboaurantium*, *S.aurantia*, and *S.inthanonensis* using both morphological and molecular evidence. *Samsoniella* is characterised by having yellow to orange, fleshy stromata and superficial perithecia and intact ascospores (Mongkolsamrit et al. 2018). Previous researchers have discovered 39 species that are mainly distributed in Asian countries such as China, Thailand, and Vietnam (Wang et al. 2024a). All *Samsoniella* species have been verified with molecular data, and a combination of six genes (ITS-nrSSU-nrLSU-*rpb1*-*rpb2*-3P*_TEF*) usually was used to study the interspecific relationship (Mongkolsamrit et al. 2018; Wang et al. 2023a, 2024a). However, the taxonomic classification of this genus is considered to be complex due to morphological plasticity, and there is a need to search for new genetic markers with higher resolution (Wang et al. 2023a).

The genus *Pleurodesmospora* was established based on *Pleurodesmosporacoccorum*, which is featured with rostella-like phialidic conidiogenous pegs pasted in erect or procumbent conidiophores (Samson and Gams 1980). *Pleurodesmospora* species are morphologically indistinguishable, emphasising the importance of molecular analysis. Based on DNA phylogeny, Chen et al. (2021a) reported that *Pleurodesmospora* belongs to Cordycipitaceae and demonstrated that the concatenated ITS-3P_*TEF* or ITS-*rpb1*-*rpb2*-3P_*TEF* datasets were reliable in studying the interspecific relationships of this genus (Chen et al. 2021a; Yeh et al. 2021). Members of *Pleurodesmospora* are known to infect various arthropods, including Araneidae, mites, leafhoppers, and whiteflies (Samson and Gams 1980; Yeh et al. 2021). To date, only five species of this genus have been described: *Pleurodesmosporacoccorum*, *P.acaricola*, *P.lemaireae*, *P.lepidopterorum*, and *P.entomophila* (Samson and Gams 1980; Chen et al. 2021a; Yeh et al. 2021; Tan and Shivas 2023, 2024). *Pleurodesmosporaacaricola*, *P.coccorum*, and *P.lepidopterorum* (Chen et al. 2021a; Yeh et al. 2021) were reported from China, and *P.lemaireae* and *P.entomophila* were found in Australia (Tan and Shivas 2023, 2024).

During the surveys of entomopathogenic fungi in Guizhou, Liaoning, and Yunnan Provinces, we have collected seven insect specimens (including six Lepidoptera and one Hymenoptera) that were infected by fungi. Based on morphology, five specimens were determined as isaria-like species, one as pleurodesmospora-like, and another one as akanthomyces-like. Further morphology studies herein and molecular phylogenetic analyses revealed four novel species belonging to *Pleurodesmospora* and *Samsoniella* and one known species of *Akanthomyces*. New findings not only enrich the species diversity of these genera but also deepen our understanding of their morphology and ecology.

## ﻿Materials and methods

### ﻿Sample collection and isolation

A survey was conducted to collect dead insect specimens with fungal infections from Guizhou, Liaoning, and Yunnan provinces (China) from July to November 2023. The specimens were collected from the lower and upper surfaces of living leaves and leaf litter on the ground in evergreen and deciduous forests with less sunlight. The fresh specimens were documented and photographed in the fields using a camera on a mobile phone. Collected specimens were placed in plastic boxes and transported to the laboratory for further examination.

To prevent contamination of fresh specimens by opportunistic fungi in the humid plastic box, fungus isolation was performed on the same day as it was collected. The fresh fruiting bodies were examined using a stereomicroscope (Olympus SZX16). A small mass of conidia on the synnemata or sclerotium inside the insect host bodies was transferred to axenic potato dextrose agar (PDA) plates using a sterile needle. The cultures were incubated at room temperature until the colonies’ size attained 2–3 cm. The pure colonies were chopped into tiny bits and stored in sterile water in a centrifuge tube and then submitted to the Kunming Institute of Botany Culture Collection (KUNCC). The fresh specimens were dried with allochroic silica gel and deposited in the Herbarium of Cryptogamic Kunming Institute of Botany Academia Sinica (HKAS), Chinese Academy of Sciences, Kunming, China.

### ﻿Morphological studies

The macro-characteristics of the fresh specimens, such as hosts, colour and shape of stroma, and the orientation of perithecia, were recorded and measured using a stereomicroscope (Leica S9E). Micro-morphological characteristics, such as perithecia, asci, ascospores, phialides, and conidia, were removed from the stromata or synnemata and mounted on a glass slide with water, lactic acid cotton blue or congo red solution. A Nikon compound microscope (Nikon ECLIPSE Ni) was used to photograph the above-mentioned microstructures. The axenic PDA plates isolated from fresh specimens were cultured at room temperature for 10–14 days, and the colony characteristics (e.g., size, shape, texture and colour) were recorded. Details of the asexual morphological characteristics from cultures were also documented with a Nikon compound microscope (Nikon ECLIPSE Ni).

### ﻿DNA extraction and polymerase chain reaction (PCR) amplification

Total genomic DNA was extracted from axenic living cultures and dry specimens using the DNA extraction kit (Omega Fungus Genomic DNA Extraction Kit, China), following the instructions of the manufacturer. Ten loci, including the internal transcribed spacers 1 and 2 along with the 5.8S rDNA (ITS), partial region of the nuclear ribosomal small subunit (nrSSU) and large subunit (nrLSU), and the largest and second-largest subunits of RNA polymerase II (*rpb1* and *rpb2*), were amplified. Several extra gene regions, including the partial region of the 3′ and the 5′ end of the translation elongation factor 1-alpha gene (3P_*TEF* and 5P_*TEF*), the replication licensing factor 7 (*MCM7*) gene, the actin beta 1 (ACT) gene and the beta-tubulin (*TUB*) gene, were amplified for *Samsoniella* species (Table 2). The primer pairs used for amplification were ITS 5 and ITS 4 for ITS (White et al. 1990), NS1 and NS4 for nrSSU (White et al. 1990), LROR and LR5 for nrLSU (Vilgalys and Hester 1990), 983F and 2218R for 3P_*TEF* (Rehner and Buckley 2005), EF1T and EF2T for 5P_*TEF* (Rehner and Buckley 2005; Bischoff et al. 2006), CRPB1A and RPB1Cr for *rpb1* (Castlebury et al. 2004), fRPB2-5f and fRPB2-7cR for *rpb2* (Castlebury et al. 2004), Mcm7-709 and Mcm7-1348rev for *MCM7* (Schmitt et al. 2009), Act-1 and Act-4R for *ACT* (Voigt and Wöstemeyer 2000), Bt2a and Bt1b for *TUB* (Glass and Donaldson 1995). All of the PCR was performed in a 25 µl reaction mixture consisting of 12.5 µl of the mixture, 7.5 µl of double distilled water, 1 µl of each primer, and 3 µl of DNA template, using a T100 Thermal Cycler (Bio-Rad). The PCR program for these six loci (nrLSU, ITS, nrSSU, 3P_*TEF*, *rpb1*, and *rpb2*) was outlined in Wei et al. (2021), while the PCR procedures for the 5P_*TEF* and *MCM7* genes were respectively given by Bischoff et al. (2006) and Schmitt et al. (2009). The PCR protocols for the *ACT* and *TUB* were respectively referenced from Voigt et al. (1999) and Glass and Donaldson (1995). The PCR products were purified and sequenced at Sangon Biotech Company (Shanghai, China) with the above-mentioned primers. The newly generated sequences were submitted to GenBank for assignment of accession number.

### ﻿Sequence alignment and phylogenetic analyses

The quality of the sequence chromatogram generated in this study was examined using BioEdit (Hall et al. 2011). The forward and reverse sequences were assembled using Seqman (Clewley 1995) and verified with those sequence data available in GenBank through the BLAST tool. Taxa used for phylogenetic analyses of Cordycipitaceae were selected following related articles (Chen et al. 2020c; Wang et al. 2020, 2024b) and BLAST research results of the newly generated sequences (Table 1).

**Table 1.** GenBank accession numbers of the taxa used in this study.

Species	strain	nrLSU	ITS	nrSSU	3P*_TEF*	*rpb1*	*rpb2*	References
*Akanthomycesaculeatus*	HUA186145^T^	MF416520			MF416465			Kepler et al. 2017
*A.aculeatus*	HUA 772	KC519370	KC519371	KC519368	KC519366			Kepler et al. 2017
*A.australiensis*	BRIP 72630a	OR527524	OR527516	OR512197	OR514840		OR514848	Kepler et al. 2017
*A.baishanensis*	CGMCC3.25673^T^	PP179404			PP464678	PP464641	PP464655	Pu et al. 2025
*A.baishanensis*	CGMCC3.25674	PP179405			PP464679	PP464642	PP464656	Pu et al. 2025
***A.baishanensis***	**HKAS144393**	**PQ492341**	**PQ492702**	**PQ492709**	**PQ499067**	**PQ499073**	**PQ499080**	**This study**
*A.bannaensis*	CLZhao 34016^T^	PP571897	PP571895				PP588774	Zhang et al. 2024
*A.buriramensis*	BCC 45158	ON008543			ON013546	ON013561		Khonsanit et al. 2024
*A.buriramensis*	BCC 47939^T^	ON008545			ON013548	ON013563		Khonsanit et al. 2024
*A.fusiformis*	BCC 40756^T^	ON008549			ON013552	ON013567	ON013576	Khonsanit et al. 2024
*A.laosensis*	YFCC 1910942	OQ509511	OQ509524		OQ506287	OQ511536	OQ511550	Wang et al. 2024b
*A.laosensis*	YFCC 1910941^T^	OQ509510	OQ509523		OQ506286	OQ511535	OQ511549	Wang et al. 2024b
*A.niveus*	BCC 79887^T^	ON008551			ON013554		ON013578	Khonsanit et al. 2024
*A.niveus*	BCC 40747	ON008550			ON013553	ON013568	ON013577	Khonsanit et al. 2024
*A.noctuidarum*	BBH 16595	MT356085	MT356073		MT477979	MT477995	MT478005	Aini et al. 2020
*A.noctuidarum*	BCC 47498	MT356086	MT356074		MT477980	MT477996	MT477988	Aini et al. 2020
*A.noctuidarum*	BCC 28571	MT356087	MT356075		MT477981	MT478009	MT478006	Aini et al. 2020
*A.noctuidarum*	BCC 36265^T^	MT356084	MT356072		MT477978	MT477994	MT477987	Aini et al. 2020
*A.phariformis*	BCC 45148^T^	ON008556			ON013559		ON013583	Khonsanit et al. 2024
*A.pseudonoctuidarum*	YFCC 1808943^T^	OQ509512	OQ509525		OQ506288	OQ511537	OQ511551	Khonsanit et al. 2024
*A.pseudonoctuidarum*	YFCC 1808944	OQ509513	OQ509526		OQ506289	OQ511538	OQ511552	Khonsanit et al. 2024
*A.pyralidarum*	BCC 32191	MT356092	MT356081		MT477983	MT478001	MT477989	Aini et al. 2020
*A.pyralidarum*	BCC 40869	MT356093	MT356082		MT477984	MT478002	MT477990	Aini et al. 2020
*A.pyralidarum*	BCC 28816^T^	MT356091	MT356080		MT477982	MT478000	MT478007	Aini et al. 2020
*Akanthomyces* sp.	BCC 76537	ON008557	ON006550		ON013560		ON013584	Aini et al. 2020
*A.taiwanicus*	NTUPPMCC 20-060	MT974356	MT974202		MW200213	MW200221	MW200230	Chuang et al. 2024
*A.tortricidarum*	BCC 28583	MT356090	MT356079		MT477986	MT477999	MT477993	Aini et al. 2020
*A.tortricidarum*	BCC 41868	MT356089	MT356077		MT477985	MT477998	MT478008	Aini et al. 2020
*A.tortricidarum*	BCC 72638^T^	MT356088	MT356076		MT478004	MT477997	MT477992	Aini et al. 2020
*A.tuberculatus*	BCC 16819	GQ249987	GQ250012	GQ249962	GQ250037			Kepler et al. 2017
*A.xixiuensis*	XX21081764^T^	OP693480	OP693460	OP693478	OP838887	OP838889	OP838891	Liu et al. 2024
*A.xixiuensis*	HKAS125851	OP693481	OP693461	OP693479	OP838888	OP838890	OP838892	Liu et al. 2024
*Arachnidicolaaraneicola*	GY 29011		MK942435			MK955945	MK955948	Chen et al. 2019
*Ara.araneogenus*	GZUIF DX1		KU893152			MH978181	MH978184	Chen et al. 2018
*Ara.bashanensis*	CQ 05621^T^	OQ300420	OQ300412		OQ325024		OQ349684	Chen et al. 2023a
*Ara.bashanensis*	CQ 05622	OQ300421	OQ300411		OQ325025		OQ349685	Chen et al. 2023a
*Ara.beibeiensis*	CQ 05921^T^	OQ300424	OQ300415		OQ325028		OQ349688	Chen et al. 2023a
*Ara.beibeiensis*	CQ 05922	OQ300427	OQ300416		OQ325029		OQ349689	Chen et al. 2023a
*Ara.coccidioperitheciatus*	NHJ 6709	EU369042	JN049865	EU369110	EU369025	EU369067	EU369086	Kepler et al. 2017
*Ara.kanyawimiae*	TBRC 7242	MF140718	MF140751		MF140838	MF140784	MF140808	Mongkolsamrit et al. 2018
*Ara.kanyawimiae*	TBRC 7244^T^	MF140716	MF140752		MF140836			Mongkolsamrit et al. 2018
*Ara.kanyawimiae*	TBRC 7243	MF140717	MF140750		MF140837	MF140783	MF140807	Mongkolsamrit et al. 2018
*Ara.kunmingensis*	YFCC 1808940^T^	OQ509509	OQ509522		OQ506285	OQ511534	OQ511548	Wang et al. 2024b
*Ara.kunmingensis*	YFCC 1808939	OQ509508	OQ509521		OQ506284	OQ511533	OQ511547	Wang et al. 2024b
*Ara.subaraneicola*	YFCC 2107937^T^	OQ509514	OQ509527		OQ506290	OQ511539	OQ511553	Wang et al. 2024b
*Ara.subaraneicola*	YFCC 2107938	OQ509515	OQ509528		OQ506291	OQ511540	OQ511554	Wang et al. 2024b
*Ara.sulphureus*	TBRC 7248^T^	MF140722	MF140758		MF140843	MF140787	MF140812	Mongkolsamrit et al. 2018
*Ara.thailandicus*	TBRC 7245^T^	MF140719	MF140754		MF140839		MF140809	Mongkolsamrit et al. 2018
*Ara.tiankengensis*	KY 11571^T^	ON502825	ON502848		ON525447		ON525446	Chen et al. 2023a
*Ara.tiankengensis*	KY 11572	ON502827	ON502821		ON525449		ON525448	Chen et al. 2023a
*Ara.waltergamsii*	TBRC 7252^T^	MF140714	MF140748		MF140834	MF140782	MF140806	Mongkolsamrit et al. 2018
*Beauveriabassiana*	ARSEF 1564		HQ880761		HQ880974	HQ880833	HQ880905	Rehner et al. 2011
*B.caledonica*	ARSEF 2567^T^	AF339520	HQ880817	NG064865	EF469057	EF469086	HQ880961	Rehner et al. 2011
*B.medogensis*	BUB 426	MG642846	MG642832	MG642889	MG642904	MG642859	MG642874	Imoulan et al. 2016
*B.scarabaeidicola*	ARSEF 5689	AF339524	JN049827	AF339574	DQ522335	DQ522380	DQ522431	Kepler et al. 2017
*B.sinensis*	BUB 504	MG642838	MG642825	MG642880	MG642895	MG642852	MG642865	Chen et al. 2013
*Cordycepsamoene-rosea*	CBS 107.73^T^	MF416550	MH860646	AY526464	MF416494	MF416651	MF416445	Wang et al. 2020
*C.amoene-rosea*	CBS 729.73	MF416551	MH860794	MF416604	MF416495	MF416652	MF416446	Wang et al. 2020
*C.coleopterorum*	CBS 110.73^T^	JF415988	AY624177	JF415965	JF416028	JN049903	JF416006	Kepler et al. 2017
*C.farinosa*	CBS 111113	MF416554	AY624181	AY526474	MF416499	MF416656	MF416450	Kepler et al. 2017
*C.fumosorosea*	CBS 244.31	MF416557	MH855200	MF416609	MF416503	MF416660	MF416454	Kepler et al. 2017
*C.javanica*	CBS 134.22	MF416558	MH854719	MF416610	MF416504	MF416661	MF416455	Kepler et al. 2017
*C.militaris*	OSC 93623	AY184966	JN049825	AY184977	DQ522332	DQ522377		Kepler et al. 2017
*C.tenuipes*	ARSEF 5135	JF415980	AY624196	MF416612	JF416020	JN049896	JF416000	Kepler et al. 2017
*Kanoksriazaquensis*	HMAS 246917	MT789696	MT789698	MT789700	MT797811	MT797809		Wang et al. 2023b
*Kanoksriazaquensis*	HMAS 246915^T^	MT789697	MT789699	MT789701	MT797812	MT797810		Wang et al. 2023b
*Lecanicilliumaraneosus*	KY 11341^T^	ON502832	ON502826		ON525443		ON525442	Chen et al. 2022
*L.araneosus*	KY 11342	ON502837	ON502844		ON525445		ON525444	Chen et al. 2022
*L.attenuatus*	CBS 402.78	AF339565	AJ292434	AF339614	EF468782	EF468888	EF468935	Kepler et al. 2017
*L.lecanii*	CBS 102067^T^	KM283795	MH862778	KM283771	KM283818	KM283838	KM283860	Kepler et al. 2017
*L.lepidopterorum*	SD05152		MT705974				MT727045	Chen et al. 2020a
*L.longisporum*	CBS 126.27^T^	KM283797	AJ292385		KM283820	KR064300	KM283862	Kepler et al. 2017
*L.muscarius*	MFLU 181145	MH497224	MH497223	MH497222	MH511807		MH511806	Kepler et al. 2017
*L.neoaraneogenus*	GZU1031Lea^T^			KX845705	KX845697	KX845699	KX845701	Shrestha et al. 2019
*L.neocoleopterorum*	GY11242		MN093297		MN097815	MN097817	MN097814	Shrestha et al. 2019
*L.pissodis*	CBS 118231^T^	KM283799		KM283775	KM283822	KM283842	KM283864	Chen et al. 2020a
*L.sabanensis*	JCh041			KC633263	KC633274			Kepler et al. 2017
*Lecanicillium* sp.	YFCC 945		OQ509531		OQ506294	OQ511543	OQ511557	Wang et al. 2024b
*L.uredinophilum*	KACC 44082^T^	KM283782		KM283758	KM283806	KM283828	KM283848	Wang et al. 2020
*L.uredinophilum*	KUN 101466	MG948307	MG948305	MG948309	MG948315	MG948311	MG948313	Wang et al. 2020
*Pleurodesmosporaacaricola*	R. Kirschner 4968		MZ435417		LC629776			Yeh et al. 2021
*P.coccorum*	CBS 460.73	MH872455	MH860743					Yeh et al. 2021
*P.entomophila*	BRIP 72652a^T^	OR527526	OR527518		OR514842		OR514850	Tan and Shivas 2023
*P.lemaireae*	BRIP 76543a^T^	PQ792647	PQ806958					Tan and Shivas 2024
*P.lepidopterorum*	DY10502		MW826577		MW834319		MW834318	Chen et al. 2021a
*P.lepidopterorum*	DY10501^T^		MW826576		MW834317	MW834315	MW834316	Chen et al. 2021a
***P.sanduensis***	**HKAS144399^T^**	**PQ492342**	**PQ492703**	**PQ492710**	**PQ499068**	**PQ499074**	**PQ499081**	**This study**
*Samsoniellaalboaurantium*	CBS 262.58^T^	MG665232	AY624179		JQ425685			Mongkolsamrit et al. 2018
*S.alboaurantium*	CBS 240.32	JF415979	AY624178		JF416019	JN049895	JF415999	Mongkolsamrit et al. 2018
*S.alpina*	YFCC 5818	MN576809		MN576753	MN576979	MN576869	MN576923	Wang et al. 2020
*S.alpina*	YFCC 5831	MN576810		MN576754	MN576980	MN576870	MN576924	Wang et al. 2020
*S.anhuiensis*	RCEF2830^T^	OM268848		OM268843	OM483864	OM751889		Wang et al. 2024a
*S.anhuiensis*	RCEF2590	OR978316		OR978313	OR966516	OR989964		Wang et al. 2024a
*S.antleroides*	YFCC 6113	MN576804		MN576748	MN576974	MN576864	MN576918	Wang et al. 2020
*S.antleroides*	YFCC 6016^T^	MN576803		MN576747	MN576973	MN576863	MN576917	Wang et al. 2020
*S.aranea*	RCEF2831	OM268849		OM268844	OM483865	OM751882	OM802500	Wang et al. 2024a
*S.aranea*	RCEF2868	OM268850		OM268845	OM483866	OM751883	OM802501	Wang et al. 2024a
*S.asiatica*	YFCC 869^T^		OQ476473		OQ506153	OQ506195	OQ506187	Wang et al. 2023a
*S.asiatica*	YFCC 870		OQ476474		OQ506154	OQ506196	OQ506188	Wang et al. 2023a
*S.asiatica*	YFCC 871		OQ476475		OQ506155	OQ506197	OQ506189	Wang et al. 2023a
*S.aurantia*	TBRC 7271	MF140728	MF140764		MF140846	MF140791	MF140818	Mongkolsamrit et al. 2018
*S.aurantia*	TBRC 7272	MF140727	MF140763		MF140845		MF140817	Mongkolsamrit et al. 2018
*S.cardinalis*	YFCC 5830	MN576788		MN576732	MN576958	MN576848	MN576902	Wang et al. 2020
*S.cardinalis*	YFCC 6144^T^	MN576786		MN576730	MN576956	MN576846	MN576900	Wang et al. 2020
*S.coccinellidicola*	YFCC 8772^T^	ON621670		ON563166	ON676514	ON676502	ON568685	Wang et al. 2022
*S.coccinellidicola*	YFCC 8773	ON621671		ON563167	ON676515	ON676503	ON568686	Wang et al. 2022
*S.coleopterorum*	A19501^T^		MT626376		MN101586	MT642600	MN101585	Chen et al. 2020c
*S.cristata*	YFCC 6023	MN576792	OQ476480	MN576736	MN576962	MN576852	MN576906	Wang et al. 2020
*S.cristata*	YFCC 7004^T^	MN576793	OQ476481	MN576737	MN576963	MN576853	MN576907	Wang et al. 2020
*S.duyunensis*	DY09162	OQ363114	OQ379242		OQ398146			Chen et al. 2023b
*S.duyunensis*	DY07501	OR263307	OR263188		OR282780	OR282773	OR282776	Chen et al. 2023b
*S.duyunensis*	DY09502	OR263427	OR263189		OR282781		OR282777	Chen et al. 2023b
*S.erucae*	KY 11121^T^	ON502835	ON502828		ON525425		ON525424	Chen et al. 2022
*S.erucae*	KY 11122	ON502822	ON502847		ON525427		ON525426	Chen et al. 2022
*S.farinospora*	YFCC 8774^T^	ON621672		ON563168	ON676516	ON676504	ON568687	Wang et al. 2022
*S.farinospora*	YFCC 9051	ON621673		ON563169	ON676517	ON676505	ON568688	Wang et al. 2022
*S.fusiformispora*	RCEF5406	OM268851		OM268846		OM751890		Wang et al. 2024a
*S.fusiformispora*	RCEF2588^T^	OR978315		OR978312				Wang et al. 2024a
*S.guizhouensis*	KY 11161^T^	ON502830	ON502823		ON525429		ON525428	Chen et al. 2022
*S.guizhouensis*	KY 11162	ON502846	ON502845		ON525431		ON525430	Chen et al. 2022
*S.haniana*	YFCC 8769^T^	ON621674		ON563170	ON676518	ON676506	ON568689	Wang et al. 2022
*S.haniana*	YFCC 8770	ON621675		ON563171	ON676519	ON676507	ON568690	Wang et al. 2022
*S.haniana*	YFCC 8771	ON621676		ON563172	ON676520	ON676508	ON568691	Wang et al. 2022
*S.hepiali*	Cor-4	MN576799		MN576743	MN576969	MN576859	MN576913	Wang et al. 2020
*S.hepiali*	YFCC 661	MN576795		MN576739	MN576965	MN576855	MN576909	Wang et al. 2020
*S.hepiali*	ICMM 82-2^T^	MN576794		MN576738	MN576964	MN576854	MN576908	Wang et al. 2020
*S.hymenopterorum*	A19521		MN128224		MN101588	MT642603		Chen et al. 2020c
*S.hymenopterorum*	A19522^T^		MN128081		MN101591	MN101589		Chen et al. 2020c
*S.inthanonensis*	TBRC 7915	MF140725	MF140761		MF140849	MF140790	MF140815	Mongkolsamrit et al. 2018
*S.kunmingensis*	YHH 16002^T^	MN576802		MN576746	MN576972	MN576862	MN576916	Wang et al. 2020
*S.lanmaoa*	YFCC 6193	MN576790		MN576734	MN576960	MN576850	MN576904	Wang et al. 2020
*S.lanmaoa*	YFCC 6148^T^	MN576789		MN576733	MN576959	MN576849	MN576903	Wang et al. 2020
*S.lasiocampidarum*	NTUPPMCC 20-061	MT974364	MT974211		MW200220	MW200229		Chuang et al. 2024
*S.lasiocampidarum*	NTUPPMCC 20-062 ^T^	MT974361	MT974208		MW200218	MW200227	MW200236	Chuang et al. 2024
*S.lasiocampidarum*	NTUPPMCC 20-063	MT974363	MT974210		MW200219		MW200238	Chuang et al. 2024
*S.*	DL 10071^T^		MN128076			MN101592		Chen et al. 2020c
*S.*	DL 10072		MN128084					Chen et al. 2020c
***S.lurida***	**HKAS144387^T^**	**PQ492339**	**PQ492700**	**PQ492707**	**PQ499065**		**PQ499078**	**This study**
***S.lurida***	**HKAS144388**	**PQ492340**	**PQ492701**	**PQ492708**	**PQ499066**	**PQ499072**	**PQ499079**	**This study**
*S.neopupicola*	KY 11322	ON502833	ON502834		ON525435		ON525434	Chen et al. 2022
*S.neopupicola*	KY 11321^T^	ON502839	ON502843		ON525433		ON525432	Chen et al. 2022
*S.pseudogunnii*	GY 407202	MZ831865	MZ831863		MZ855234		MZ855240	Chen et al. 2021b
*S.pseudogunnii*	GY 407201	MZ827010	MZ827470		MZ855233		MZ855239	Chen et al. 2021b
*S.pseudotortricidae*	YFCC 9052^T^	ON621677		ON563173	ON676521	ON676509	ON568692	Wang et al. 2022
*S.pseudotortricidae*	YFCC 9053	ON621678		ON563174	ON676522	ON676510	ON568693	Wang et al. 2022
*S.pupicola*	DY 101682	MZ827635	MZ827008		MZ855232		MZ855238	Chen et al. 2021b
*S.pupicola*	DY 101681^T^	MZ827009	MZ827085		MZ855231		MZ855237	Chen et al. 2021b
*S.ramosa*	YFCC 6020^T^	MN576805		MN576749	MN576975	MN576865	MN576919	Wang et al. 2020
*S.sanmingense*	CGMCC3.25661	PP179392		PP177395	PP482033	PP464664	PP464647	Pu et al. 2025
*S.sanmingense*	CGMCC3.25662 ^T^	PP179393		PP177396	PP482034	PP464665	PP464648	Pu et al. 2025
*S.sapaensis*	YFCC 873^T^		OQ476489		OQ506152	OQ506194	OQ506186	Wang et al. 2023a
*S.sapaensis*	YFCC 872		OQ476488		OQ506151	OQ506193	OQ506185	Wang et al. 2023a
*S.*	YFCC 8766^T^	ON621679		ON563175	ON676523	ON676511	ON568694	Wang et al. 2022
*S.*	YFCC 8767	ON621680		ON563176	ON676524	ON676512	ON568695	Wang et al. 2022
*S.*	YFCC 8768	ON621681		ON563177	ON676525	ON676513	ON568696	Wang et al. 2022
***S.subasiatica***	**HKAS144400^T^**	**PQ492343**	**PQ492704**	**PQ492711**	**PQ499069**	**PQ499075**	**PQ499082**	**This study**
*S.tiankengensis*	KY 11741^T^	ON502838	ON502840		ON525437		ON525436	Chen et al. 2022
*S.tiankengensis*	KY 11742	ON502841	ON502849		ON525439		ON525438	Chen et al. 2022
*S.tortricidae*	YFCC 6013	MN576807		MN576751	MN576977	MN576867	MN576921	Wang et al. 2020
*S.tortricidae*	YFCC 6142	MN576808		MN576752	MN576978	MN576868	MN576922	Wang et al. 2020
*S.tortricidae*	YFCC 6131^T^	MN576806		MN576750	MN576976	MN576866	MN576920	Wang et al. 2020
***S.torquatistipitata***	**HKAS144411^T^**	**PQ492345**	**PQ492706**	**PQ492713**	**PQ499071**	**PQ499077**	**PQ499084**	**This study**
***S.torquatistipitata***	**HKAS144402**	**PQ492344**	**PQ492705**	**PQ492712**	**PQ499070**	**PQ499076**	**PQ499083**	**This study**
*S.vallis*	DY091092	OR263431	OR263190		OR282783			Chen et al. 2023b
*S.vallis*	DY091091	OR263428	OR263191		OR282782			Chen et al. 2023b
*S.vallis*	DY07242	OR263308	OR263186		OR282779		OR282775	Chen et al. 2023b
*S.vallis*	DY07241^T^	OR263306	OR263159		OR282778	OR282772	OR282774	Chen et al. 2023b
*S.winandae*	MY12469.01^T^	OM491231	OM491228		OM687896	OM687901	OM687899	Crous et al. 2023b
*S.yuanzuiensis*	NTUPPMCC 20-064^T^	MT974359	MT974206			MW200225	MW200234	Chuang et al. 2024
*S.yuanzuiensis*	NTUPPMCC 20-065	MT974360	MT974207		MW200217	MW200226	MW200235	Chuang et al. 2024
*S.yunnanensis*	YFCC 1527^T^	MN576812		MN576756	MN576982	MN576872	MN576926	Wang et al. 2020
*S.yunnanensis*	YFCC 1824	MN576813		MN576757	MN576983	MN576873	MN576927	Wang et al. 2020
*S.yunnanensis*	YFCC 7282	MN576814		MN576758	MN576984	MN576874	MN576928	Wang et al. 2020
*Simplicilliumlanosoniveum*	CBS 101267	AF339554	AJ292395		DQ522357	DQ522405	DQ522463	Spatafora et al. 2007
*Sim.lanosoniveum*	CBS 704.86	AF339553			DQ522358	DQ522406	DQ522464	Spatafora et al. 2007

Note: Types are indicated by T. The newly generated sequences in this study were shown in bold.

In order to investigate the interspecific relationship among *Samsoniella*, a separated phylogenetic analysis based on combined four-gene (5P_*TEF*+3P_*TEF*+*rpb1*+*MCM7*) was performed with a larger taxa sampling from this genus (Table 2). The four loci were independently aligned with reference sequences using MAFFT v.7 (http://mafft.cbrc.jp/alignment/server/). The alignments of each locus were improved using Trimal v.1.2 (Capella-Gutiérrez et al. 2009) and were concatenated using Sequence Matrix v. 1.7.8 (Vaidya et al. 2011). The final combined dataset was converted to a NEXUS file for Bayesian inference analysis and a FASTA file for maximum likelihood analysis using Aliview (Larsson 2014).

**Table 2.** GenBank accession numbers of the *Samsoniella* used in this study.

Species	strain	3P_*TEF*	5P_*TEF*	*rpb1*	*MCM7*	References
*Samsoniellaalboaurantium*	CBS 240.32	JF416019		JN049895		Mongkolsamrit et al. 2018
*S.alboaurantium*	CBS 262.58^T^	MF416497		MF416654		Mongkolsamrit et al. 2018
*S.alpina*	YFCC 5818^T^	MN576979	OQ506160	MN576869	OQ506229	Wang et al. 2023a
*S.alpina*	YFCC 5831	MN576980	OQ506161	MN576870	OQ506230	Wang et al. 2023a
*S.antleroides*	YFCC 6016^T^	MN576973	OQ506162	MN576863	OQ506231	Wang et al. 2023a
*S.antleroides*	YFCC 6113	MN576974	OQ506163	MN576864	OQ506232	Wang et al. 2023a
*S.anhuiensis*	RCEF2830^T^	OM483864		OM751889		Wang et al. 2024a
*S.anhuiensis*	RCEF2590	OR966516		OR989964		Wang et al. 2024a
*S.aranea*	RCEF2831	OM483865		OM751882		Wang et al. 2024a
*S.aranea*	RCEF2868	OM483866		OM751883		Wang et al. 2024a
*S.asiatica*	YFCC 869^T^	OQ506153	OQ506164	OQ506195	OQ506233	Wang et al. 2023a
*S.asiatica*	YFCC 870	OQ506154	OQ506165	OQ506196	OQ506234	Wang et al. 2023a
*S.asiatica*	YFCC 871	OQ506155	OQ506166	OQ506197	OQ506235	Wang et al. 2023a
*S.aurantia*	TBRC 7271^T^	MF140846		MF140791		Mongkolsamrit et al. 2018
*S.aurantia*	YFCC 874	OQ506157	OQ506167	OQ506199	OQ506236	Wang et al. 2023a
*S.aurantia*	YFCC 880	OQ506156	OQ506168	OQ506198	OQ506237	Wang et al. 2023a
*S.cardinalis*	YFCC 5830	MN576958	OQ506169	MN576848	OQ506238	Wang et al. 2023a
*S.cardinalis*	YFCC 6144^T^	MN576956	OQ506170	MN576846	OQ506239	Wang et al. 2023a
*S.coccinellidicola*	YFCC 8772^T^	ON676514		ON676502		Wang et al. 2022
*S.coccinellidicola*	YFCC 8773	ON676515		ON676503		Wang et al. 2022
*S.*	A19501^T^	MN101586		MT642600		Chen et al. 2020c
*S.cristata*	YFCC 6023	MN576962	OQ506171	MN576852	OQ506240	Wang et al. 2023a
*S.cristata*	YFCC 7004^T^	MN576963	OQ506172	MN576853	OQ506241	Wang et al. 2023a
*S.duyunensis*	DY09162	OQ398146				Chen et al. 2023b
*S.duyunensis*	DY07501	OR282780		OR282773		Chen et al. 2023b
*S.duyunensis*	DY09502	OR282781				Chen et al. 2023b
*S.erucae*	KY11121^T^	ON525425				Chen et al. 2022
*S.erucae*	KY11122	ON525427				Chen et al. 2022
*S.farinospora*	YFCC 8774^T^	ON676516		ON676504		Wang et al. 2022
*S.farinospora*	YFCC 9051	ON676517		ON676505		Wang et al. 2022
*S.fusiformispora*	RCEF5406			OM751890		Wang et al. 2024a
*S.guizhouensis*	KY11161^T^	ON525429				Chen et al. 2022
*S.guizhouensis*	KY11162	ON525431				Chen et al. 2022
*S.haniana*	YFCC 8769^T^	ON676518		ON676506		Wang et al. 2022
*S.haniana*	YFCC 8771	ON676520		ON676508		Wang et al. 2022
*S.hepiali*	ICMM 82-2^T^	MN576964	OQ506173	MN576854	OQ506242	Wang et al. 2023a
*S.hepiali*	YFCC 868	OQ506158	OQ506175	OQ506200	OQ506244	Wang et al. 2023a
*S.hepiali*	YFCC 2702	MN576966	OQ506174	MN576856	OQ506243	Wang et al. 2023a
*S.hymenopterorum*	A19521	MN101588		MT642603		Chen et al. 2020c
*S.hymenopterorum*	A19522^T^	MN101591		MN101589		Chen et al. 2020c
*S.inthanonensis*	TBRC 7915^T^	MF140849		MF140790		Mongkolsamrit et al. 2018
*S.kunmingensis*	YHH 16002^T^	MN576972		MN576862		Wang et al. 2023a
*S.lanmaoa*	YFCC 6148^T^	MN576959	OQ506176	MN576849	OQ506245	Wang et al. 2023a
*S.lanmaoa*	YFCC 6193	MN576960	OQ506177	MN576850	OQ506246	Wang et al. 2023a
*S.lasiocampidarum*	NTUPPMCC 20-061	MW200220		MW200229		Chuang et al. 2024
*S.lasiocampidarum*	NTUPPMCC 20-062^T^	MW200218		MW200227		Chuang et al. 2024
*S.lasiocampidarum*	NTUPPMCC 20-063	MW200219				Chuang et al. 2024
*S.*	DL 10071^T^			MN101592		Chen et al. 2020c
***S.lurida***	**HKAS144387^T^**	**PQ499065**				**This study**
***S.lurida***	**HKAS144388**	**PQ499066**		**PQ499072**	**PV158406**	**This study**
*S.neopupicola*	KY11321^T^	ON525433				Chen et al. 2022
*S.neopupicola*	KY11322	ON525435				Chen et al. 2022
*S.pseudogunnii*	GY407201^T^	MZ855233				Chen et al. 2021b
*S.pseudogunnii*	GY407202	MZ855234				Chen et al. 2021b
*S.pseudotortricidae*	YFCC 9052^T^	ON676521		ON676509		Wang et al. 2022
*S.pseudotortricidae*	YFCC 9053	ON676522		ON676510		Wang et al. 2022
*S.pupicola*	DY101681^T^	MZ855231				Chen et al. 2021b
*S.pupicola*	DY101682	MZ855232				Chen et al. 2021b
*S.ramosa*	YFCC 6020^T^	MN576975	OQ506178	MN576865		Wang et al. 2023a
*S.sanmingense*	CGMCC3.25661	PP482033		PP464664		Pu et al. 2025
*S.sanmingense*	CGMCC3.25662	PP482034		PP464665		Pu et al. 2025
*S.sapaensis*	YFCC 872	OQ506151	OQ506179	OQ506193	OQ506247	Wang et al. 2023a
*S.sapaensis*	YFCC 873^T^	OQ506152	OQ506180	OQ506194	OQ506248	Wang et al. 2023a
*S.*	YFCC 8766^T^	ON676523		ON676511		Wang et al. 2022
*S.*	YFCC 8767	ON676524		ON676512		Wang et al. 2022
***S.subasiatica***	**HKAS144400^T^**	**PQ499069**	**PV158402**	**PQ499075**	**PV158407**	**This study**
*S.tiankengensis*	KY11741^T^	ON525437				Chen et al. 2022
*S.tiankengensis*	KY11742	ON525439				Chen et al. 2022
*S.tortricidae*	YFCC 6131^T^	MN576976	OQ506181	MN576866	OQ506249	Wang et al. 2023a
*S.tortricidae*	YFCC 6142	MN576978	OQ506182	MN576868	OQ506250	Wang et al. 2023a
***S.torquatistipitata***	**HKAS144411^T^**	**PQ499071**		**PQ499077**	**PV158408**	**This study**
***S.torquatistipitata***	**HKAS144402**	**PQ499070**		**PQ499076**	**PV158409**	**This study**
*S.vallis*	DY091092	OR282783				Chen et al. 2023b
*S.vallis*	DY091091	OR282782				Chen et al. 2023b
*S.vallis*	DY07242	OR282779				Chen et al. 2023b
*S.vallis*	DY07241^T^	OR282778		OR282772		Chen et al. 2023b
*S.winandae*	MY12469.01^T^	OM687896		OM687901		Crous et al. 2023b
*S.yuanzuiensis*	NTUPPMCC 20-064^T^			MW200225		Chuang et al. 2024
*S.yuanzuiensis*	NTUPPMCC 20-065	MW200217		MW200226		Chuang et al. 2024
*S.yunnanensis*	YFCC 1527^T^	MN576982	OQ506183	MN576872	OQ506251	Wang et al. 2020, 2023a
*S.yunnanensis*	YFCC 1824	MN576983	OQ506184	MN576873	OQ506252	Wang et al. 2020, 2023a
*Akanthomyceswaltergamsii*	YFCC 883	OQ506159		OQ506201	OQ506253	Wang et al. 2023a

Note: Types are indicated by T. The newly generated sequences in this study were shown in bold.

Maximum likelihood (ML) analysis was performed using IQ-TREE 1.6.12 (Minh et al. 2020) with branch support being estimated from 1000 ultrafast bootstraps. The Bayesian inference (BI) analysis was run on MrBayes on XSEDE (3.2.7a) in the CIPRES Science Gateway. The GTR+I+G model was selected as the best-fit substitution model by MrModeltest 2.3 implemented in MrMTgui v.1.0 (Nylander 2004; Nuin 2007). Four simultaneous Markov chains were run for 100,000,000 generations, and trees were sampled every 1000 generations. Finally, phylogenetic trees were visualised using Figtree v.1.4.0 (Rambaut 2016) and edited using Adobe Illustrator 2020.

## ﻿Results

### ﻿Phylogenetic analyses

**Figure 1.**
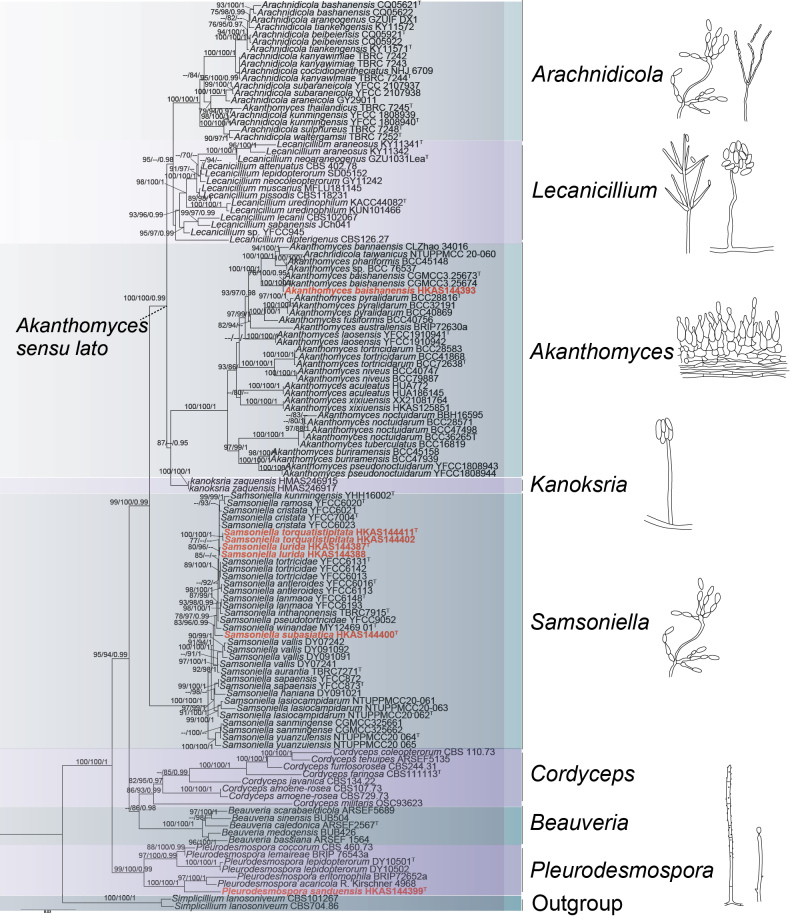
Phylogram generated from maximum likelihood analysis of Cordycipitaceae based on a six-locus dataset (nrLSU, ITS, nrSSU, 3P*_TEF*, *rpb1* and *rpb2*). SH-aLRT support ≥ 75%, ultrafast bootstrap support (UFB) ≥ 75%, and PP values ≥ 95% are indicated above or below branches. A hyphen (–) indicates values lower than 75% SH-aLRT, 75% UFB, and 95% PP. The isolates in this study are shown in bold red. Generic names are indicated on the right side of the tree. Ex-types are indicated by “T”.

The six-locus dataset (nrLSU, ITS, nrSSU, *3P_TEF*, *rpb1*, and *rpb2*) comprises 118 representative taxa sampled from nine genera within Cordycipitaceae, with two strains of *Simplicilliumlanosoniveum* (CBS 101267 and CBS 704.86) selected as the outgroup. The ML tree inferred from the six-locus dataset is shown in Fig. 1, in which the seven strains generated in this study belong to three genera: *Akanthomyces*, *Pleurodesmospora* and *Samsoniella*. The isolate HKAS144393 clusters with *Akanthomycesbaishanensis* (CGMCC3.25673 and CGMCC3.25674) with strong statistical support (100% SH-aLRT / 100% UFB / 1.00 PP, Fig. 1). The isolate HKAS144399 constitutes a distinct lineage which branches off the clade of *Pleurodesmosporaacaricola* and *P.entomophila* with maximum support (100% SH-aLRT / 100% UFB / 1.00 PP, Fig. 1). The remaining five strains (HKAS144411, HKAS144402, HKAS144388, HKAS144402, and HKAS144400) group with species of *Samsoniella* with inadequate support.

**Figure 2.**
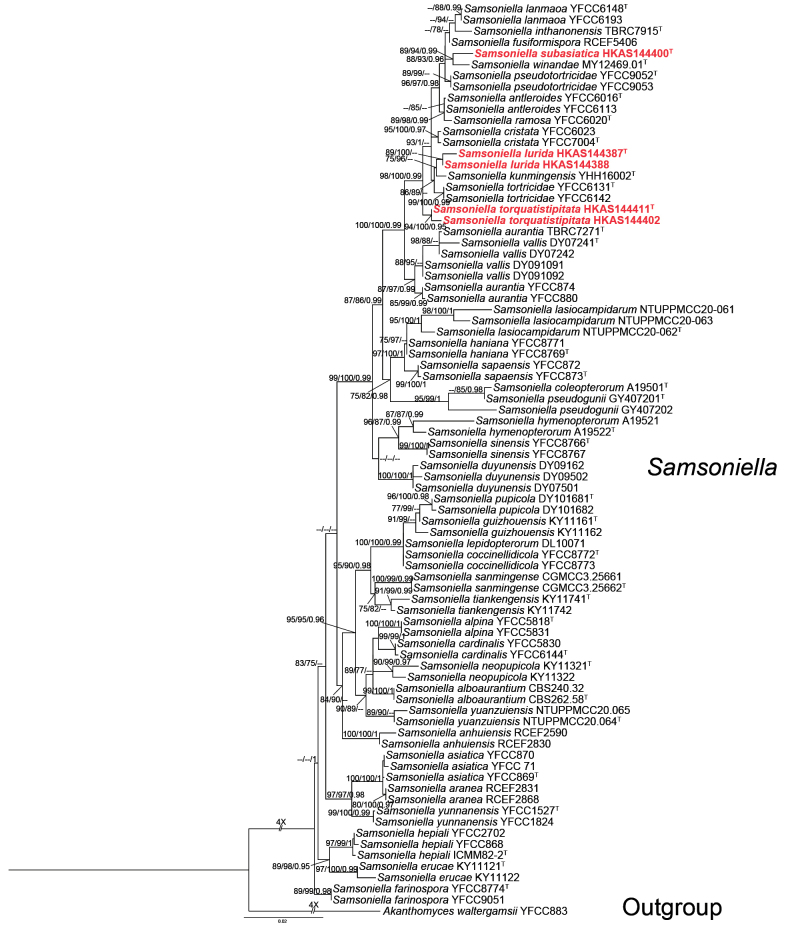
Phylogram generated from maximum likelihood analysis of *Samsoniella* based on a four-locus dataset (5P_*TEF*+3P_*TEF*+*rpb1*+*MCM7*). SH-aLRT support ≥ 75%, ultrafast bootstrap support ≥ 75%, and PP values ≥ 95% are indicated above or below branches. A hyphen (–) indicates values lower than 75% SH-aLRT, 75% UFB, and 95% PP. The isolates in this study are shown in bold red. Ex-types are indicated by “T”.

To clarify the phylogenetic placements of the five specimens of *Samsoniella*, a separated phylogenetic tree based on four genes (5P_*TEF*+3P_*TEF*+*rpb1*+*MCM7*) was constructed with larger taxa sampling from *Samsoniella*. The four-locus dataset included 79 taxa of *Samsoniella* with 3077 bp characters (737 bp for 5P_*TEF*, 986 bp for nrSSU, 725 bp for 3P*_TEF*, 629 bp for *rpb1*). *Akanthomyceswaltergamsii* YFCC 883 was designated as the out-group taxon. The ML tree (Fig. 2) shows that the isolates HKAS144387 and HKAS144388 are sisters to *S.kunmingensis* and are closely related to *S.tortricidae*, with moderate support (86% SH-aLRT / 89% UFB, Fig. 2). The isolate HKAS144400 shows a sister relationship to *Samsoniellawinandae* with significant support (89% SH-aLRT / 94% UFB / 0.99 PP, Fig. 2). The isolates HKAS144411 and HKAS144402 were placed in a clade distantly related to other *Samsoniella* species with strong support (98% SH-aLRT / 100% UFB / 1.00 PP, Fig. 2). The guidelines of Maharachchikumbura et al. (2021) were followed when determining whether species were novel.

### ﻿Taxonomy

#### ﻿ Akanthomyces baishanensis

Taxon classificationFungiSordariomycetesCordycipitaceae

H.L. Pu & J.Z. Qiu, in Pu, Yang, Keyhani, Yang, Zheng, Qiu, Mao, Shang, Lin, Xiong, Lin, Lai, Huang, Yuan, Liang, Fan, Ma, Qiu & Qiu, J. Fungi 11(1, no. 28): 16 (2025)

B28DB3A4-0AA1-5E2A-863C-CFF6B8D50000

Index Fungorum: IF903210

Fig. 3

##### Description.

**Figure 3.**
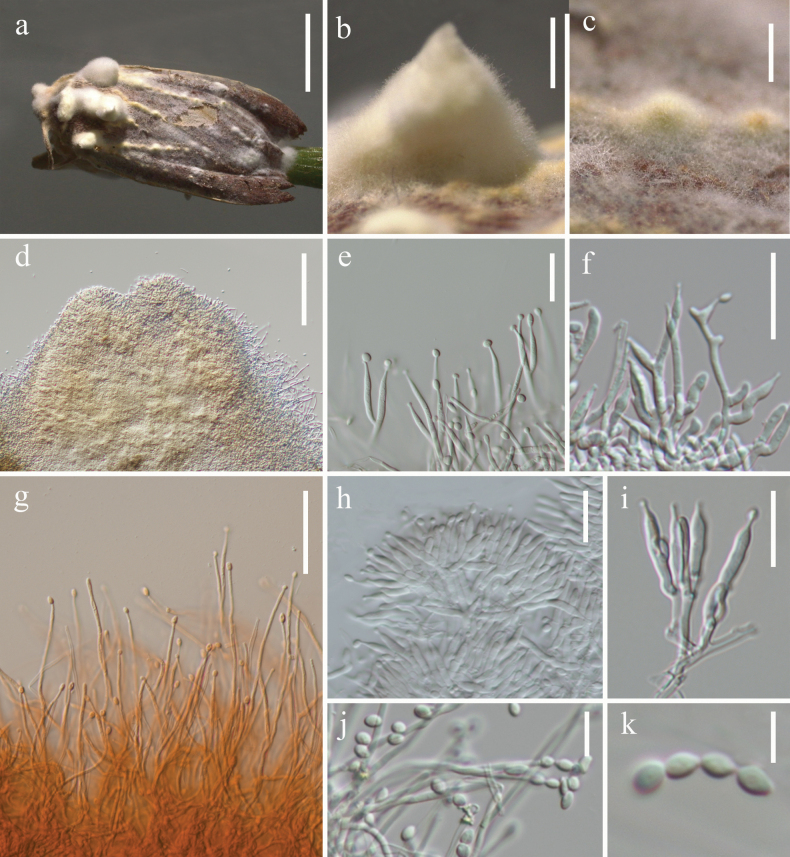
*Akanthomycesbaishanensis* (HKAS144393) **a** fungus on an adult moth **b–d** synnemata **e–k** phialides and conidia. Scale bars: 5 mm (**a**); 1 mm (**b**); 0.5 mm (**c**); 100 µm (**d**); 30 µm (**g**); 20 µm (**e, f, h, i**); 10 µm (**j**); 5 µm (**k**).

Parasitic on moth (Lepidoptera). **Sexual morph.** See Pu et al. (2025). **Asexual morph. *Synnemata*** arising from the moth body, white, erect, simple, subuliform (2 × 2.7 mm) or subglobose (0.2 × 0.5 mm). ***Hyphae*** smooth, septate, hyaline, 1.4–2.5 μm (*x̄* = 1.8 µm, n = 30) in diam. ***Conidiophores*** developing from superficial hyphae of synnemata, micronematous, branched, smooth-walled, bearing solitary to clusters of phialides. ***Phialides*** 6–29.6 × 1.6–3.2 µm (*x̄* = 19 × 2.7 µm, n = 30), monophialidic, trimorphic, arising from anastomosing mycelia, slender filiform in shape (Fig. 3G), or arising from conidiophores, cylindrical (Fig. 3E, H, I) or subuliform (Fig. 3F) at basal portion, tapering into a thin neck. ***Conidia*** 3.2–4.7 × 1.8–2.8 µm (*x̄* = 3.9 × 2.2 µm, n = 50), forming on tip of phialides, hyaline, smooth-walled, fusiform, globose or broadly ovoid, gathering in chains.

##### Material examined.

China • Liaoning Province, Tieling City (42°17'22.3"N, 123°50'22.2"E), on a dead adult moth (Lepidoptera) on the stem of a plant, 25 August 2023, Ting-Chi Wen, HLJ2023082515 (HKAS144393).

##### Notes.

Phylogenetic analysis based on six gene markers revealed that the specimen HKAS144393 and *Akanthomycesbaishanensis* (CGMCC3.25673 and CGMCC3.25674) form a robustly supported monophyletic clade (100% SH-aLRT / 100% UFB / 1.00 PP, Fig. 1). Both HKAS144393 and *A.baishanensis* exhibit parasitic relationships with adult moths. Notably, HKAS144393 represents a naturally occurring asexual morph characterised by trimorphic conidiogenous structures, while the asexual morph of *A.baishanensis* described by Pu et al. (2025) was obtained from culture and displayed only a single type of conidiogenous structure. Our observations demonstrate greater morphological plasticity in this species than previously recognised.

#### ﻿ Pleurodesmospora sanduensis

Taxon classificationFungiSordariomycetesCordycipitaceae

J. Bu, K.D. Hyde & T.C. Wen sp. nov.

D5B197EF-6B67-5009-86DD-63F04AF46381

Index Fungorum: IF903211

Fig. 4

##### Etymology.

In reference to the location of the type specimen, Sandu County of Guizhou Province, China.

##### Description.

Parasitic on adult Lepidoptera. **Sexual morph.** Undetermined. **Asexual morph. *Colonies*** on natural specimen white, sparse, only covering the abdomen of host. ***Conidiophores*** micronematous, cylindrical, erect or procumbent, sparsely branched, smooth, hyaline, septate, ca. 1.3–2.8 μm (*x̄* = 2 µm, n = 30) in width, from the middle part to the distal end densely covered by numerous minute, dentiform pegs, 0.7–1.8 × 0.5–0.8 µm (*x̄* = 1 × 0.7 µm, n = 25). ***Conidia*** obovoid, globose, smooth-walled, 2.7–4.8 × 1.4–2.5 µm (*x̄* = 3.7 × 2 µm, n = 30), arranged in short chains.

**Figure 4.**
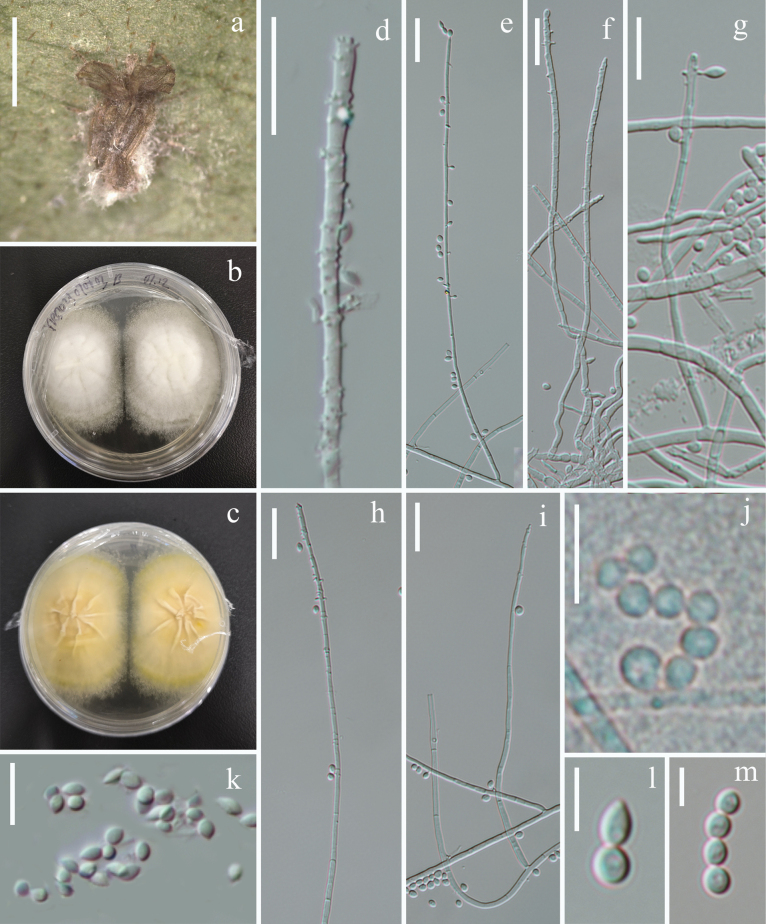
*Pleurodesmosporasanduensis* (HKAS144399) **a** fungus on host **b, c** obverse **(b)** and reverse **(c)** of colony on PDA**d–I** conidiophore and conidiogenous cells **j, k–m** conidia adhering in a chain. Scale bars: 2 mm (**a**); 20 µm (**d, e, f, h, i**); 10 µm (**g, j, k**); 5 µm (**l, m**).

##### Culture characteristics.

colonies on PDA reaching a diameter of 42 mm in three weeks at room temperature, white, circular, velvety, flat, edge entire, surface wrinkled, with radially striate, mycelia dense at centre, becoming loose outward, reverse cream-yellow.

##### Type.

China • Guizhou Province, Qiannan Buyei and Miao Autonomous Prefecture, Sandu County, the Yaoren Mountain (25°59'41"N, 107°56'41"E, alt. 987.1 m), on a dead adult of Lepidoptera on leaf litter, 08 July 2023, Jing Bu, YRS23070803B (holotype HKAS144399, ex-holotype KUNCC24-18538).

##### Notes.

Six-locus phylogenetic analyses show that the *Pleurodesmosporasanduensis* is separated from other species of *Pleurodesmospora* with strong statistical support (100% SH-aLRT / 100% UFB / 1.00 PP, Fig. 1). *Pleurodesmosporasanduensis* is phylogenetically closely related to *P.acaricola* and *P.entomophila*. Pairwise nucleotide differences between *P.sanduensis* and *P.entomophila* (Tan and Shivas 2023) revealed 6 bp in nrLSU, 28 bp in ITS, 25 bp in *3P_TEF*, and 74 bp in *rpb2*. These molecular divergences support the recognition of *P.sanduensis* as a novel species, consistent with the taxonomic thresholds proposed by Jeewon and Hyde (2016). *Pleurodesmosporasanduensis* is similar to *P.acaricola* in producing loose and white colonies covering the host. However, *Pleurodesmosporasanduensis* differs from *P.acaricola* by its larger conidia (2.7–4.8 × 1.4–2.5 µm vs. 2.5–3 × 2 µm) in chains, but it is solitary in *P.acaricola* (Yeh et al. 2021). Additionally, chlamydospores are observed in *P.acaricola*, while it is absent in *P.sanduensis*.

#### ﻿ Samsoniella lurida

Taxon classificationFungiSordariomycetesCordycipitaceae

J. Bu, K.D. Hyde & T.C. Wen sp. nov.

5E4D7BB2-219E-5087-A878-FCC83306D325

Index Fungorum: IF903212

Fig. 5

##### Etymology.

Referring to the pale stromata arising from the host, which is different from other species in *Samsoniella*.

**Figure 5.**
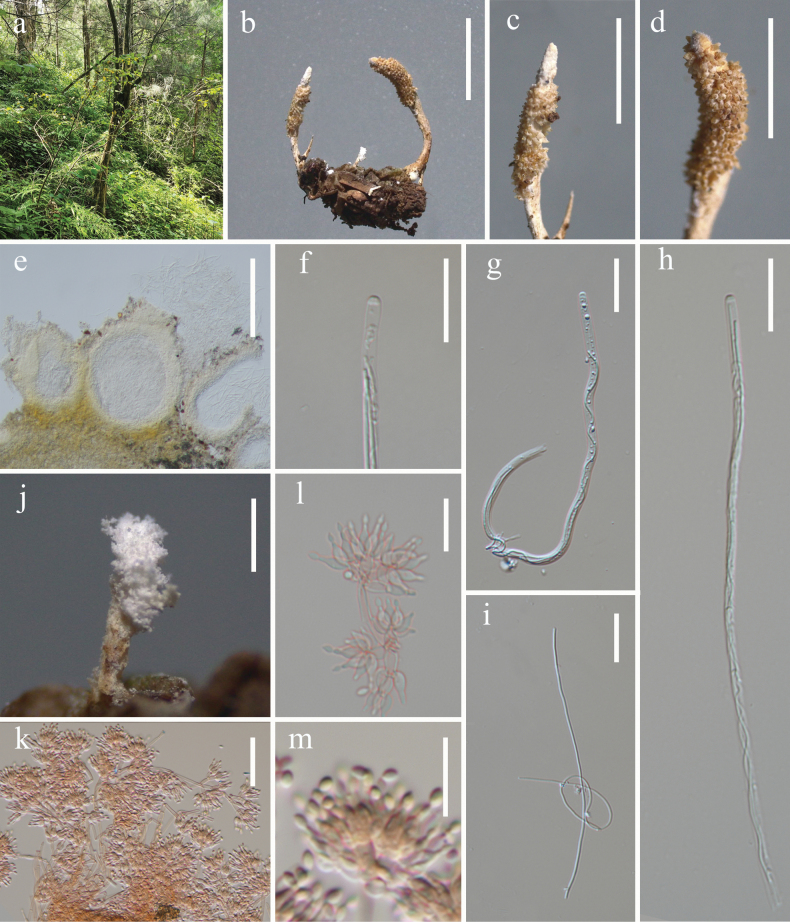
*Samsoniellalurida* (HKAS144387) **a** habitat **b** stromata and synnemata arising from host **c, d** fertile part with perithecia **e** vertical section of perithecia **f** ascus cap **g, h** asci **i** ascospore **j** synnema **k–m** conidiophores, phialides and conidia. Scale bars: 5 mm (**b**); 3 mm (**c, d**); 200 µm (**e**); 20 µm (**f, g, h, i**); 0.5 mm (**j**); 30 µm (**k**); 10 µm (**l, m**).

##### Description.

Parasitic on cocoon of Lepidoptera. **Sexual morph. *Stromata*** 6.4–8.6 mm long, pale orange, cylindrical, unbranched or branched at base, arising from the head and end of the insect cocoon. ***Stipe*** cylindrical, pale orange, 0.4–0.8 mm wide. ***Fertile part*** clavate, pale orange, 2.5–3.1 × 0.6–1 mm, often with sterile tip (0.5–1.2 mm). The lateral sides had a longitudinal ditch without perithecia. ***Perithecia*** superficial, crowded, broadly ovoid, 205–455 × 144–274 µm (*x̄* = 319 × 198 µm, n = 15). ***Asci*** hyaline, cylindrical, 128–219 × 1.4–3.6 µm (*x̄* = 170 × 2.6 µm, n = 20). ***Ascus caps*** hemispherical, hyaline, 1.2–1.8 × 1.6–3 μm (*x̄* = 1.5 × 2.5 μm, n = 20). ***Ascospores*** filiform, hyaline, aseptate, 86–175 × 0.4–1 μm (*x̄* = 132 × 0.7 μm, n = 15) wide, do not disarticulate into part-spores. **Asexual morph. *Synnemata*** arising from the middle of the host, erect, single, 1.2 × 0.2–0.35 mm, producing a mass of floccose conidia at the apex. ***Hyphae*** smooth-walled, hyaline, septate, 1.5–3.6 µm (*x̄* = 2.5 µm, n = 30) wide. ***Conidiophores*** smooth-walled, cylindrical, verticillate, 2.3–9.1 × 1.9–2.9 µm (*x̄* = 4.9 × 2.3 µm, n = 15). ***Phialides*** verticillate, in whorls of two to five, lageniform, 4.2–7.3 µm (*x̄* = 5.7 µm, n = 30) long, basal portion cylindrical, tapering abruptly toward the apex, from 1.7–2.5 µm (*x̄* = 2.1 µm, n = 30) wide (base) to 0.5–0.9 µm (*x̄* = 0.7 µm, n = 30) wide (apex). ***Conidia*** smooth-walled, hyaline, fusiform, 1.9–2.7 × 1.1–1.9 µm (*x̄* = 2.3 × 1.4 µm, n = 30).

##### Type.

China • Yunnan Province, Kunming City, Panlong District, the Longchuanqiao Forest Park (25°17'05.26"N, 102°78'07.88"E, alt. 1963.9 m), on a lepidopteran cocoon buried in soil, 20 September 2023, Jing Bu, LCQ2023092034B (holotype HKAS144387).

##### Additional materials examined.

China • Yunnan Province, Kunming, Xishan District, Tuanjie Country (25°08'61.38"N, 102°46'11.71"E, alt. 1971.2 m) on lepidopteran larva buried in soil, 17 October 2023, Jing Bu, MLSX2023101741B (HKAS144388, living culture KUNCC24-18534).

##### Notes.

Phylogenetic analyses revealed that two specimens of *Samsoniellalurida* (HKAS144387 and HKAS144388) are closely related to *S.kunmingensis* and *S.tortricidae* (Fig. 2). Morphological comparisons demonstrate distinct characteristics among these species. *S.kunmingensis* and *S.tortricidae* produce larger, brightly coloured, multi-branched stromata with oblong-ovate to fusiform perithecia; *S.lurida* is characterised by pallid stromata and broadly ovoid perithecia (Table 3). Furthermore, *S.lurida* possesses a unique sterile tip, a feature not observed in other known *Samsoniella* species. Sequence comparisons between *S.lurida* and *S.kunmingensis* showed that there are 8 bp differences within 943 bp 3P_*TEF* and 12 bp differences within 979 bp *rpb2*. *S.lurida* differs from *S.tortricidae* by 10 bp within 943 bp 3P_*TEF* and 11 bp within 979 bp *rpb2*. Both morphological characters and molecular analyses support this fungus as a new species in *Samsoniella* (Jeewon and Hyde 2016).

**Table 3.** Comparison between the sexual morphs in *Samsoniella*. The data generated in this study are shown in bold.

Species	Host	Stromata (mm)	Fertile Part (mm)	Perithecia (µm)	Asci (µm)	Ascospores (µm)	References
*S.cristata*	Lepidopteran pupa	solitary or two, 25–40 long, crista-like	crista-like or subulate, 3.1–18.5 × 0.9–8.0	superficial, narrowly ovoid, 370–485 × 150–245	cylindrical,8-spored,180–356 × 3.0–4.8	bola-shaped, septate, 155–290 × 1.0–1.3	Wang et al. 2020
*S.inthanonensis*	Lepidopteran larva	gregarious, 20–50 long, 1–1.5 broad, cylindrical to clavate	clavate, 8–15 × 1.5–2	superficial, ovoid, 417.5–474.5 × 205–260	cylindrical, 8-spored, 300 × 2–2.5	bola-shaped, 3 or 4 septate, 221.5–267 × 0.5–1	Mongkolsamrit et al. 2018
*S.kunmingensis*	Lepidopteran pupa	solitary, 23 long, cylindrical to clavate	clavate, 3.3–4.2 × 0.8–1.2	superficial, narrowly ovoid to fusiform, 330–395 × 110–185	cylindrical, 8-spored, 150–297 × 3.0–4.6	bola-shaped, septate, 127–190 × 0.8–1.5	Wang et al. 2020
*S.lanmaoa*	Lepidopteran pupa	two to five, 38–69 long, palmately branched	clavate, 8.5–11.2 × 0.6–2.3	superficial, narrowly ovoid to fusiform, 360–467 × 124–210	cylindrical, 8-spored, 160–325 × 3.3–4.8	bola-shaped, septate, 135–260 × 0.9–1.4	Wang et al. 2020
***S.lurida***	**Lepidopteran pupa**	**6.4–8.6 long, cylindrical**	**clavate, 2.5–3.1 × 0.6–1.0, sterile tip 0.5–1.2 wide**	**superficial, broadly ovoid, 205–455 × 144–274**	**cylindrical, 128–219 × 1.4–3.6**	**filiform, aseptate, 86.1–174.7 × 0.4–1.0**	**This study**
*S.pseudotortricidae*	Lepidopteran pupa	solitary to several, 20–65 long, clavate	clavate to subulate, 10–17 × 1.5–4.2	superficial, narrowly ovoid to fusiform, 285.7–313.2 × 149.2–154.9	/	/	Wang et al. 2022
***S.torquatistipitata***	**Coleoptera**	**solitary, 4.4 × 0.1–0.3, clavate**	**clavate, 1.5 × 0.4**	**superficial, lageniform, 263–353 × 174–238**	**cylindrical, 8-spored, up to 114–173 × 1.6–3.3**	**filiform, 86.2–125.7 × 0.3–0.6**	**This study**
*S.tortricidae*	Lepidopteran cocoon	gregarious, 25–60	clavate to subulate, 5–15 × 1.2–2.3	superficial, narrowly ovoid to fusiform, 350–468 × 140–225	cylindrical, 8-spored, 170–285 × 2.8–4.0	bola-shaped, septate, 120–235 × 0.8–1.3	Wang et al. 2020
*S.winandae*	Lepidopteran cocoon	multiple, 8–20 long and 0.5–2 broad, cylindrical to enlarging apically	clavate, 2–8 × 2–3	superficial, narrowly ovoid, 500–570 × 135–180	cylindrical, 8-spored, 300 × 4–5	bola shaped, 3 or 5 septate, 200–265 × 0.5–1	Crous et al. 2023b

#### ﻿ Samsoniella torquatistipitata

Taxon classificationFungiSordariomycetesCordycipitaceae

J. Bu, K.D. Hyde & T.C. Wen sp. nov.

BC17707F-4005-5A0E-B464-CD6462B8A70E

Index Fungorum: IF903213

Fig. 6

##### Etymology.

From the Latin “torqu”, referring to the stipe of stroma, is torsional rather than cylindrical.

**Figure 6.**
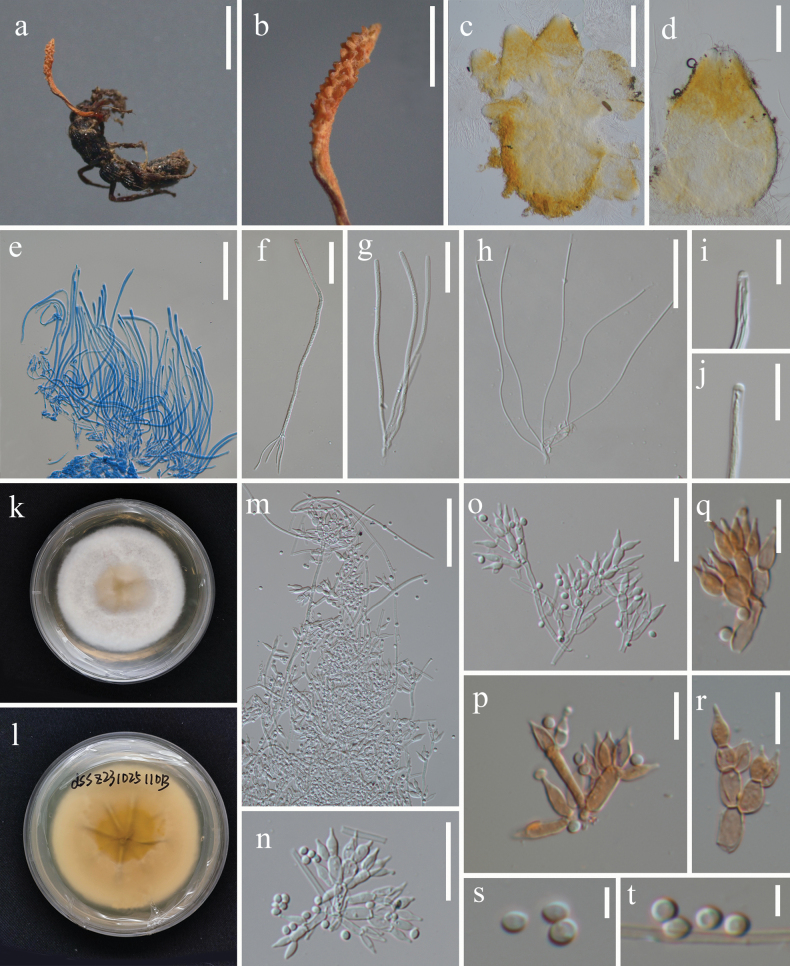
*Samsoniellatorquatistipitata* (HKAS144411) **a** fungus on the adult ant **b** fertile part **c** vertical section of stroma **d** perithecium **e–g** asci **h** ascospore **i, j** ascus cap **k, l** obverse (**k**) and reverse (**l**) of colony on PDA; **m–r** conidiophores and phialides; **s, t** conidia. Scale bars: 3 mm (**a**); 1 mm (**b**); 200 µm (**c**); 100 µm (**d**); 50 µm (**e, m**); 30 µm (**f, g, h**); 20 µm (**n, o**); 10 µm (**i, j, p, q, r**); 3 µm (**s, t**).

##### Description.

Parasitic on ant (Hymenopteran). **Sexual morph. *Stroma*** arising from head of ant, orange, single, simple, 4.4 × 0.1–0.3 mm. ***Stipe*** fleshy, torsional, reddish-orange, up to 2.7 mm long. ***Fertile part*** cylindrical, becoming acuate toward the end, reddish-orange, 1.7 × 0.4 mm. ***Perithecia*** lageniform, superficial, 255–368 × 163–244 µm (*x̄* = 288 × 190 µm, n = 5), growing on one side of fertile part. ***Asci*** cylindrical, hyaline, 8-spored, 114–173 × 1.6–3.3 µm (*x̄* = 135 × 2.4 µm, n = 20), with hemispherical cap, 1.7–2.5 × 1.1–1.8 µm (*x̄* = 2.2 × 1.4 µm, n = 20). ***Ascospores*** filiform, aseptate, hyaline, 86–125 × 0.3–0.6 μm (*x̄* = 98.6 × 0.5 µm, n = 15), non-disarticulating. **Asexual morph**. produced on the cultures, hyphomycetous. ***Hyphae*** smooth, septate, hyaline, 1.2–2.0 μm (*x̄* = 1.6 µm, n = 30) in diam. ***Conidiophores*** smooth-walled, cylindrical or elongated ellipsoid, verticillate with phialides in whorls of two to five or singly along the hyphae, 4.4–18.4 × 1.7–3.9 µm (*x̄* = 8.4 × 2.7 µm, n = 30). ***Phialides*** lageniform, 6.1–10.7 µm (*x̄* = 8.0 µm, n = 30) long, basal portion inflated, 1.8–3.5 µm (*x̄* = 2.6 µm, n = 30) wide, tapering abruptly into a thin neck, 0.7–1.4 µm (*x̄* = 0.9 µm, n = 30) wide. ***Conidia*** subglobose, hyaline, 1.8–2.8 µm (*x̄* = 2.3 µm, n = 50) in diam.

##### Culture characteristics.

colonies on PDA reaching 40 mm in 14 days at room temperature, circular, flat, edge entire, mycelia dense, cottony, creamy yellow at centre, becoming white outward, with concentric rings, sporulation, reverse creamy yellow, with radially striate.

##### Type.

China • Yunnan Province, Puer City, Simao District, Plum Lake Park (22°72'66.83"N, 100°97'83.57"E, alt. 1354.5 m), on an adult ant (Hymenoptera) buried in soil, 25 October 2023, Jing Bu, DSSZ20231025110B (holotype HKAS144411, ex-holotype KUNCC24-18535).

##### Additional materials examined.

China • Yunnan Province, Puer, Simao District, Plum Lake Park (22°75'14.29"N, 100°97'73.13"E, alt. 1338.8 m), on lepidopteran cocoon buried in soil, 26 October 2023, Jing Bu, MZH20231025119B (paratype HKAS144402, ex-paratype KUNCC24-18536).

##### Notes.

The phylogenetic tree (Fig. 2) showed that *Samsoniellatorquatistipitata* constitutes a distinct clade distantly related to *S.cristata*, *S.kunmingensis*, *S.lurida*, and *S.tortricidae*. A pairwise comparison of *3P_TEF*, *rpb1*, *MCM7*, and *rpb2* showed that *S.torquatistipitata* differs from *S.cristata*, *S.kunmingensis*, *S.lurida*, and *S.tortricidae* in 1–6 bp, 3–4 bp, 6–9 bp, and 4–16 bp, respectively. *Samsoniellatorquatistipitata* is characterised by the small, single stroma (4.4 mm long), reddish-orange, cylindrical fertile part, superficial, lageniform perithecia, and the association with adult ants. Morphological comparisons of the novel taxa with closely related *Samsoniella* species are provided in Table 3. Both morphological characteristics and molecular analyses support this fungus as a new species in *Samsoniella* (Jeewon and Hyde 2016).

#### ﻿ Samsoniella subasiatica

Taxon classificationFungiSordariomycetesCordycipitaceae

J. Bu, K.D. Hyde & T.C. Wen sp. nov.

183F34B4-F8B1-52AB-8A21-8B76D94EEE1A

Index Fungorum: IF903214

Fig. 7

##### Etymology.

Referring to the morphology similar to *Samsoniellaasiatica*.

**Figure 7.**
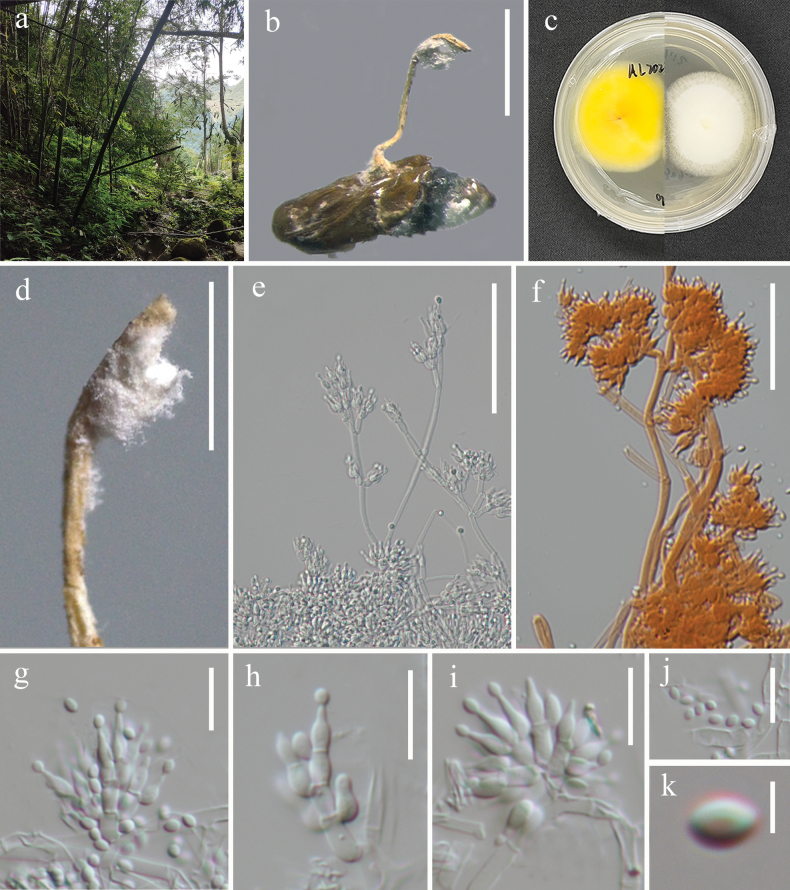
*Samsoniellasubasiatica* (HKAS144400) **a** habitat **b** synnema arising from pupa **c** lower and upper view of the colony on PDA**d** synnema **e, f** conidiophores **g–i** phialides **j, k** conidia. Scale bars: 2 mm (**b**); 1 mm (**d**); 50 µm (**e**); 30 µm (**f**); 10 µm (**g, h, i, j**); 2 µm (**k**).

##### Description.

Parasitic on pupa of Lepidoptera. **Sexual morph**. Undetermined. **Asexual morph**. ***Synnema*** arising from middle part of pupa, solitary, erect, flexuous, unbranched, 2.8 × 0.2 mm. ***Stipe*** cylindrical, pale orange. ***Hyphae*** smooth-walled, septate, hyaline 1.3–2.8 µm (*x̄* = 2.0 µm, n = 50). ***Conidiophores*** grouped together at the apex of synnema and the head of pupa, verticillate 3.6–7.4 × 2–3 µm (*x̄* = 5.2 × 2.4 µm, n = 20). ***Phialides*** lageniform, usually in whorls of two to five, 4.2–6.8 µm (*x̄* = 5.6 µm, n = 50) long, globose at basal portion, tapering gradually toward the apex, from 1.8–2.4 µm (*x̄* = 2.1 µm, n = 50) wide (base) to 0.6–1 µm (*x̄* = 0.8 µm, n = 50) wide (apex). ***Conidia*** single, smooth-walled, hyaline, fusiform to oval, 1.9–2.9 × 1.4–1.8 μm (*x̄* = 2.4 × 1.6 µm, n = 50).

##### Culture characteristics.

Colonies on PDA reaching a diameter of 27–29 mm in two weeks at room temperature, white, circular, velvety, mycelia dense, becoming loose in the outmost ring, reverse brightly yellow.

##### Type.

China • Guizhou Province, Qiannan Buyei and Miao Autonomous Prefecture, Anlong County (24°99'08.43"N, 105°59'76.06"E, alt. 1395.6 m), on lepidopteran pupa on leaf litter, 07 September 2023, Jing Bu, Al2023090717B (holotype HKAS144400, ex-holotype KUNCC24-18537).

##### Notes.

*Samsoniellasubasiatica* morphologically resembles *S.asiatica* (Wang et al. 2023a) by producing a flexuous synnema, pale orange stipe, with a mass of conidia at the apex. However, *S.subasiatica* differs from *S.asiatica* in having simple synnema and larger conidia (1.9–2.9 μm vs. 1.1–1.8 μm) (Table 4). The synnema of *S.asiatica* is branched at the base (Wang et al. 2023a). Furthermore, phylogenetic analysis based on four loci revealed that *S.subasiatica* is sister to *S.winandae*, with moderate statistical support (89% SH-aLRT / 94% UFB / 0.99 PP; Fig. 2). However, *S.subasiatica* can be distinguished from *S.winandae* by its significantly smaller synnemata and phialides (4.2–6.8 × 1.8–2.4 µm vs. 5–12 × 2–3 µm) (Table 4). Additionally, a comparison of nucleotide sequences between *S.subasiatica* and *S.winandae* indicated that there are 6 bp differences in *3P_TEF*, 14 bp in *rpb1*, and 8 bp in *rpb2*. Based on the recommendations made by Jeewon and Hyde (2016), we determined this fungus as a novel species.

**Table 4.** Comparison between the asexual morphs in *Samsoniella*. The data generated in this study are shown in bold.

Species	Host	Synnemata (mm)	Conidiophores (µm)	Phialides	Phialides Size (µm)	Conidia (µm)	References
*S.aurantia*	Lepidopteran larva	25–75 × 1–1.5	150 × 2–3	/	(5–)7.5(–9) × 2–3	fusiform, oval with pointed ends, (2–)2.5(–3) × 1–2	Mongkolsamrit et al. 2018
*S.asiatica*	Lepidopteran pupa	4–26 × 0.4–1.5	4.6–10.3 × 0.8–1.9	verticillate, in whorls of two to four, or solitary on hyphae	2.7–8.6 × 0.7–1.7, 0.6–1.1 wide at apex	fusiform or oval, 1.1–1.8 × 0.8–1.2	Wang et al. 2023a
*S.cristata*	Lepidopteran pupa	/	3.6–11.5 × 1.7–2.5	verticillate, in whorls of two to five, or solitary on hyphae	4.5–23.2 × 1.6–2.7, 0.5–1.1 wide at apex	fusiform or oval, 2.4–3.2 × 1.6–2.3	Wang et al. 2020
*S.inthanonensis*	Lepidopteran larva	/	2–3 wide	verticillate, in whorls of two to five, cylindrical basal portion	basal (4–)6.5–10(–12) × (1–)1.5–2(3), neck (1–)2.5(–4) × 0.5–1	fusiform, (2–)3(–3.5) × 1.5–2	Mongkolsamrit et al. 2018
*S.lanmaoa*	Lepidopteran pupa	/	3.8–13.3 × 1.5–2.1	verticillate, in whorls of two to six, usually solitary on hyphae	3.5–20.7 × 1.7–2.6, 0.5–1.1 wide at apex	fusiform or oval, 1.9–2.7 × 1.4–2.0	Wang et al. 2020
***S.lurida***	**Lepidopteran pupa**	**1.2 × 0.2–0.35**	**2.3–9.1 × 1.9–2.9**	**verticillate, in whorls of two to five**	**4.2–7.3** × **1.7–2.5, 0.5–0.9 wide at apex**	**fusiform, 1.9–2.7 × 1.1–1.9**	**This study**
*S.pseudotortricidae*	Lepidopteran pupa	/	6.6–26.5 × 1.1–2.5	verticillate, in whorls of two to five, usually solitary on hyphae	5.4–6.9 × 1.0–1.6, 0.5–0.8 wide at apex	fusiform or oval, 0.9–1.5 × 0.8–1.3	Wang et al. 2022
***S.subasiatica***	**Lepidopteran pupa**	**2.8 × 0.2**	**3.6–7.4 × 2–3**	**verticillate, in whorls of two to five**	**4.2–6.8** × **1.8–2.4, 0.6–1.0 wide at apex**	**fusiform to oval, 1.9–2.9 × 1.4–1.8**	**This study**
***S.torquatistipitata***	**Coleopteran adult**	/	**4.4–18.4 × 1.7–3.9**	/	**6.1–10.7** × **1.8–3.5, 0.7–1.4 wide at apex**	**subglobose, up to 1.8–2.8 in diameter**	**This study**
*S.vallis*	Lepidopteran pupa	/	11.3–22.1 × 1.3–1.4	single phialide or whorls of two to four	7.2–8.1 × 2.8–3.2	fusiform to ellipsoidal, 2.3–3.1 × 1.5–2.1	Chen et al. 2023b
*S.winandae*	Lepidopteran pupa and cocoon	12 × 2	/	verticillate, in whorls of two to five	5–12 × 2–3	ellipsoidal, 1.5–3 × 1–2	Crous et al. 2023b

## ﻿Discussion

### ﻿Morphology-phylogeny of *Akanthomycessensu lato*

*Akanthomycessensu lato* is a monophyletic lineage, and it was segregated into four genera, including *Akanthomycessensu stricto*, *Arachnidicola*, *Lecanicillium* and *Kanoksria*, corresponding to their morphological and ecological traits (Khonsanit et al. 2024; Wang et al. 2024b, Fig. 1). *Akanthomycessensu stricto* comprises seventeen species pathogenic to moths, characterised by white to creamy synnemata with cylindrical, papillate phialides and catenulate conidia (Aini et al. 2020; Khonsanit et al. 2024). *Arachnidicola* comprises twelve species primarily pathogenic to spiders, displaying isaria-like anamorphs (Mongkolsamrit et al. 2018; Chen et al. 2022; 2023a; Wang et al. 2024b), except for *Akanthomycesthailandicus*, which has a lecanicillium-like anamorph (Mongkolsamrit et al. 2018). *Lecanicillium* includes twelve species pathogenic to diverse hosts (e.g., Lepidoptera, Coleoptera, Hemiptera, spiders) with acremonium-like and verticillium-like anamorphs (Chiriví-Salomón et al. 2015; Chen et al. 2017, 2020a, 2020b, 2022; Manfrino et al. 2022). *Kanoksria*, a monotypic genus basal to the others, exhibits simplicillium-like anamorphs and is a hyperparasite on *Ophiocordycepssinensis* (Wang et al. 2023b).

In this study, we identified a moth-pathogenic species, *Akanthomycesbaishanensis*, which exhibits the typical phialide characteristics of *Akanthomycessensu stricto*, along with previously undescribed phialide types within this clade. Although molecular data provide precise taxonomic evidence, morphological and ecological traits remain indispensable. An integrated taxonomy approach is necessary for resolving these complex fungal groups. Furthermore, ecological features may also provide valuable insights for the identification and discovery of novel *Akanthomyces* species.

### ﻿The molecular phylogeny and morphology of *Samsoniella*

Sexual morphs of *Samsoniella* share similarities in producing yellowish to reddish-orange, fleshy, simple to branched stromata; superficial, ovoid to fusiform perithecia; cylindrical asci with thickened apex and filiform, multiseptate, non-disarticulating ascospores (Mongkolsamrit et al. 2018). Species of this genus are indistinguishable solely based on sexual morphology. However, they can be divided into two types based on their stroma size: ***Type Ia*** includes nine species with a length of stromata more than 25 mm and is pathogenic to lepidopteran hosts (Mongkolsamrit et al. 2018; Wang et al. 2020, 2022, 2023b); ***Type IIa*** includes six species with a length of stromata lower than 25 mm and are pathogenic to lepidopteran and hymenopteran hosts or hyperparasitic to *Cordyceps* species (Wang et al. 2020; Crous et al. 2023b) (Table 5). In this study, we introduce two new species in this group, namely, *Samsoniellalurida* and *S.torquatistipitata*, based on their sexual and asexual morphs. It is worth noting that *S.torquatistipitata* is pathogenic to an adult ant and has a very small, solitary, simple, reddish-orange stroma (4.4 mm in length). This is the first time to report the sexual typified species from an adult ant and contribute to the morphological diversity of *Samsoniella*.

**Table 5.** Morphological synopsis of *Samsoniella* species.

Type	Species	Morphological characteristics	Host	References
Type Ia	*S.antleroides*, *S.aurantia*, *S.cristata*, *S.inthanonensis*, *S.lanmaoa*, *S.pseudotortricidae*, *S.ramosa*, *S.sapaensis*, *S.tortricidae*.	Stromata orange, fleshy, solitary to gregarious, simple or branched, more than 25 mm in length	Lepidoptera	Mongkolsamrit et al. 2018; Wang et al. 2020, 2022, 2023b
Type IIa	*S.cardinalis*, *S.hepiali*, *S.kunmingensis*, ***S.lurida***, ***S.torquatistipitata***, *S.winandae*.	Stromata orange, fleshy, solitary to gregarious, usually unbranched, less than 25 mm in length	*Cordyceps* sp., Lepidoptera	Wang et al. 2020; Crous et al. 2023b
Type Ib	*S.asiatica*, *S.aurantia*, *S.coccinellidicola*, *S.duyunensis*, *S.erucae*, *S.haniana*, *S.lasiocampidarum*, *S.ramosa*, *S.sapaensis*, *S.subasiatica*, *S.tiankengensis*, *S.vallis*, *S.winandae*, *S.yuanzuiensis*, *S.yunnanensis*.	Synnemata erect, terminal irregularly branched, with conidial mass at the subterminal region of synnemata, conidal mass powdery and floccose	Lepidoptera, Coleoptera, Hymenoptera, *Cordyceps* sp.	Mongkolsamrit et al. 2018; Wang et al. 2020, 2022, 2023a; Chen et al. 2022, 2023b; Crous et al. 2023b; Chuang et al. 2024
Type IIb	*S.alpina*, *S.anhuiensis*, *S.aranea*, *S.hepiali*, *S.hymenopterorum*, *S.*, *S.neopupicola*, *S.pupicola*, *S.pseudogunnii*, *S.sanmingense*.	White colonies surround the host surface without synnemata	Lepidoptera, Coleoptera, Hymenoptera, Spider.	Chen et al. 2020c, 2021b, 2022; Wang et al. 2020, 2022, 2024a; Pu et al. 2025

The asexual morphs of *Samsoniella* have been known from 39 species. Macromorphologically, they can be categorised into two types: ***Type Ib*** includes 16 species which have well-developed stromata and are pathogenic to Lepidoptera, Coleoptera, Hymenoptera and *Cordyceps* sp. (Mongkolsamrit et al. 2018; Wang et al. 2020, 2022, 2023a; Chen et al. 2022, 2023b; Crous et al. 2023b; Chuang et al. 2024); ***Type IIb*** includes 15 species which form white colonies on the host surface and are pathogenic to Lepidoptera, Coleoptera, Hymenoptera, and spiders (Chen et al. 2020c, 2021b, 2022; Wang et al. 2020, 2022, 2024a). Our new species *S.subasiatica* was known only from its asexual morphs. This species has well-developed stroma covered with a white, powdery conidia mass, extremely resembling *S.asiatica*. However, these two species are phylogenetically distant, indicating that characteristics of asexual morphs have less taxonomic significance in interspecific demarcation.

Collectively, taxonomic inferences from phylogenetic analyses do not align with the morphological categories outlined in Table 5. The morphological plasticity of *Samsoniella* species limits their utility in taxonomy, necessitating molecular analyses for accurate species delineation (Mongkolsamrit et al. 2018). The six-locus (nrLSU+ITS+nrSSU+3P*_TEF*+*rpb1*+*rpb2*) phylogeny effectively resolves genetically distant species, while it struggles with closely related taxa, particularly due to the limited resolution of the ITS regions. In contrast, the four-gene (5P*_TEF*+3P*_TEF*+*rpb1*+*MCM7*, Wang et al. 2023a) dataset provides superior resolution, highlighting its importance in refining the taxonomy of *Samsoniella*.

## Supplementary Material

XML Treatment for Akanthomyces baishanensis

XML Treatment for Pleurodesmospora sanduensis

XML Treatment for Samsoniella lurida

XML Treatment for Samsoniella torquatistipitata

XML Treatment for Samsoniella subasiatica

## ﻿Additional information

### Author ORCIDs

Jing Bu https://orcid.org/0009-0006-6861-7770

De-Ping Wei https://orcid.org/0000-0002-6576-2239

Zheng-Hui Liu https://orcid.org/0000-0001-7022-4075

Zhong-Liang Liu https://orcid.org/0009-0007-9519-1418

Ji-Chuan Kang https://orcid.org/0000-0002-6294-5793

Xing-Can Peng https://orcid.org/0000-0002-7271-7639

Zhang-Jiang He https://orcid.org/0000-0002-7120-1227

Shi-Ke Huang https://orcid.org/0000-0002-2936-396X

Xian Zhang https://orcid.org/0009-0008-0919-4303

Kevin D. Hyde https://orcid.org/0000-0002-2191-0762

Nalin N. Wijayawardene https://orcid.org/0000-0003-0522-5498

Ting-Chi Wen https://orcid.org/0000-0003-1744-5869

### Data availability

All of the data that support the findings of this study are available in the main text or Supplementary Information.

## Supplementary materials

Supplementary material 1 Alignment of Cordycipitaceae tree-six locus Data type fas Explanation note The alignment of Cordycipitaceae tree that based on six locus (nrLSU, ITS, nrSSU, tef-1α, rpb1 and rpb2). https://binary.pensoft.net/file/1303958This dataset is made available under the Open Database License (http://opendatacommons.org/licenses/odbl/1.0/). The Open Database License (ODbL) is a license agreement intended to allow users to freely share, modify, and use this Dataset while maintaining this same freedom for others, provided that the original source and author(s) are credited.Jing Bu

Supplementary material 2 Alignment of Samsoniella tree-five locus Data type fas Explanation note The alignment of Samsoniella tree based on five locus (nrLSU, nrSSU, tef-1α, rpb1 and rpb2). https://binary.pensoft.net/file/1303959This dataset is made available under the Open Database License (http://opendatacommons.org/licenses/odbl/1.0/). The Open Database License (ODbL) is a license agreement intended to allow users to freely share, modify, and use this Dataset while maintaining this same freedom for others, provided that the original source and author(s) are credited.Jing Bu

Supplementary material 3 Legend for supplementary figures of single gene tree Data type docx https://binary.pensoft.net/file/1303960This dataset is made available under the Open Database License (http://opendatacommons.org/licenses/odbl/1.0/). The Open Database License (ODbL) is a license agreement intended to allow users to freely share, modify, and use this Dataset while maintaining this same freedom for others, provided that the original source and author(s) are credited.Jing Bu
