# Supplementary material for: ﻿Molecular phylogeny and morphology reveal four novel species in Cordycipitaceae in China
Source: MycoKeys. 2025 Apr 9;116:91–124. doi: 10.3897/mycokeys.116.147006 (PMC12004076; doi:10.3897/mycokeys.116.147006)
Supplement: Supplementary material 3 — Legend for supplementary figures of single gene tree [file mycokeys-116-091-s003.docx]

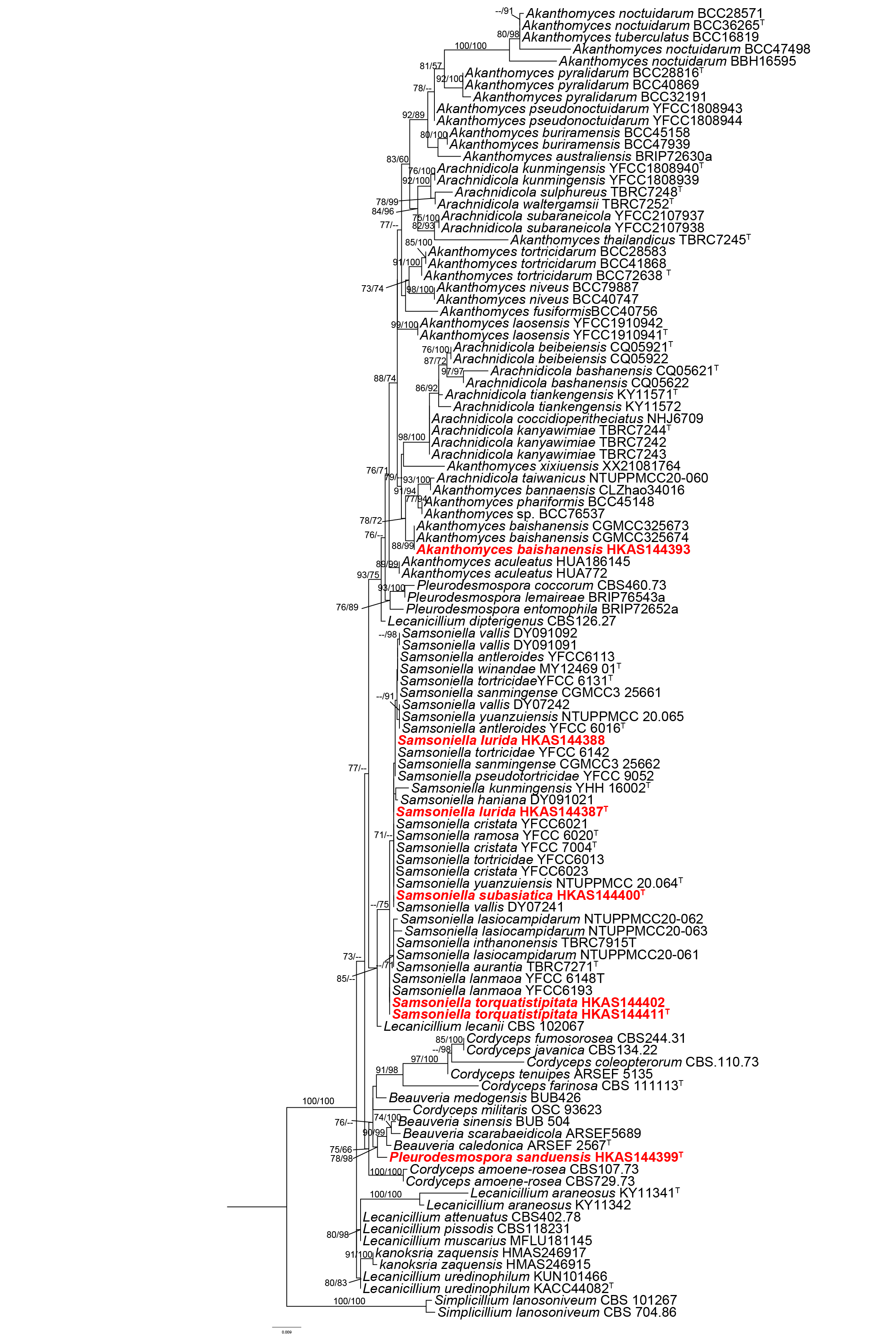


**Figure S1** The LSU-based tree in Fig. 1 of the manuscript. The strain HKAS144393 close to *Akanthomyces baishanensis* CGMCC325674 with moderate statistical support (88% SH-aLRT / 99% UFB). The five specimens of *Samsoniella* species cluster with other *Samsoniella* without significant genetic distance, indicating LSU gene has low resolution in demarcation at species level for this genus. *Pleurodesmoapora sanduensis* **sp. nov.** does not cluster with *Pleurodesmospora coccorum* CBS 460.73 and *Pleurodesmospora entomophila* BRIP 72652a. Instead, it forms a branch which is close to the genus *Beauveria* and *Cordyceps*, suggesting that LSU gene is insufficient for generic delimitation.


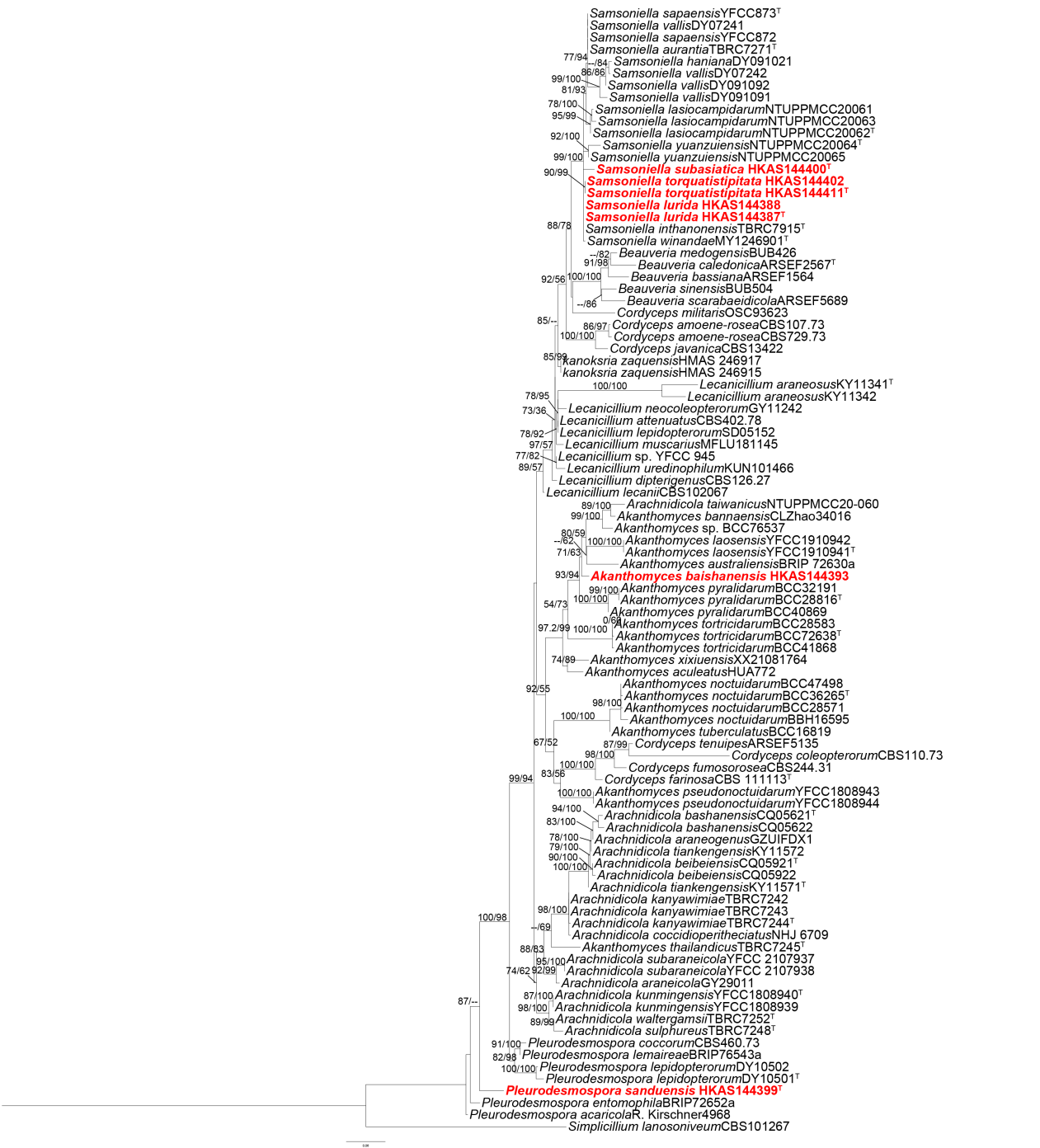


**Figure S2** The ITS-based tree in Fig. 1 of the manuscript. The seven specimens in this study can be naturally located in respective genera, indicating that ITS gene has good performance on generic demarcation. Specimens of *Samsoniella* cluster with remaining species of this genus without significant genetic distance, indicating this gene is not enough for species identification. *Pleurodesmospora sanduensis* **sp. nov.** HKAS 144399 form a distinct clade that is separated from other species of *Pleurodesmospora* with adequate support (87% SH-aLRT).


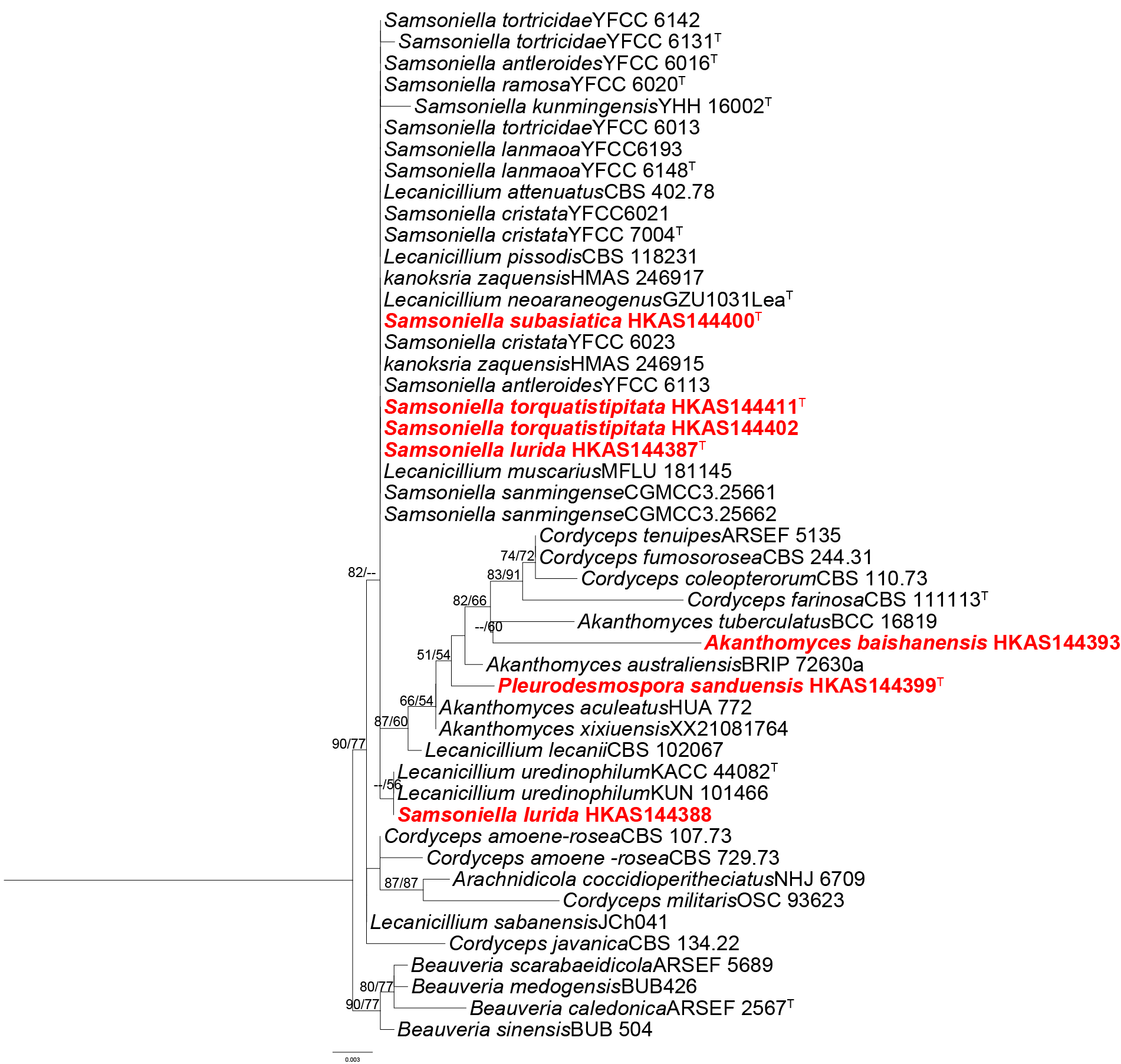


**Figure S3** The SSU-based tree in Fig. 1 of the manuscript. SSU gene is relatively conserved compared to other ones and thus SSU-based tree can not reflect the natural classification of Cordycipitaceae specie.


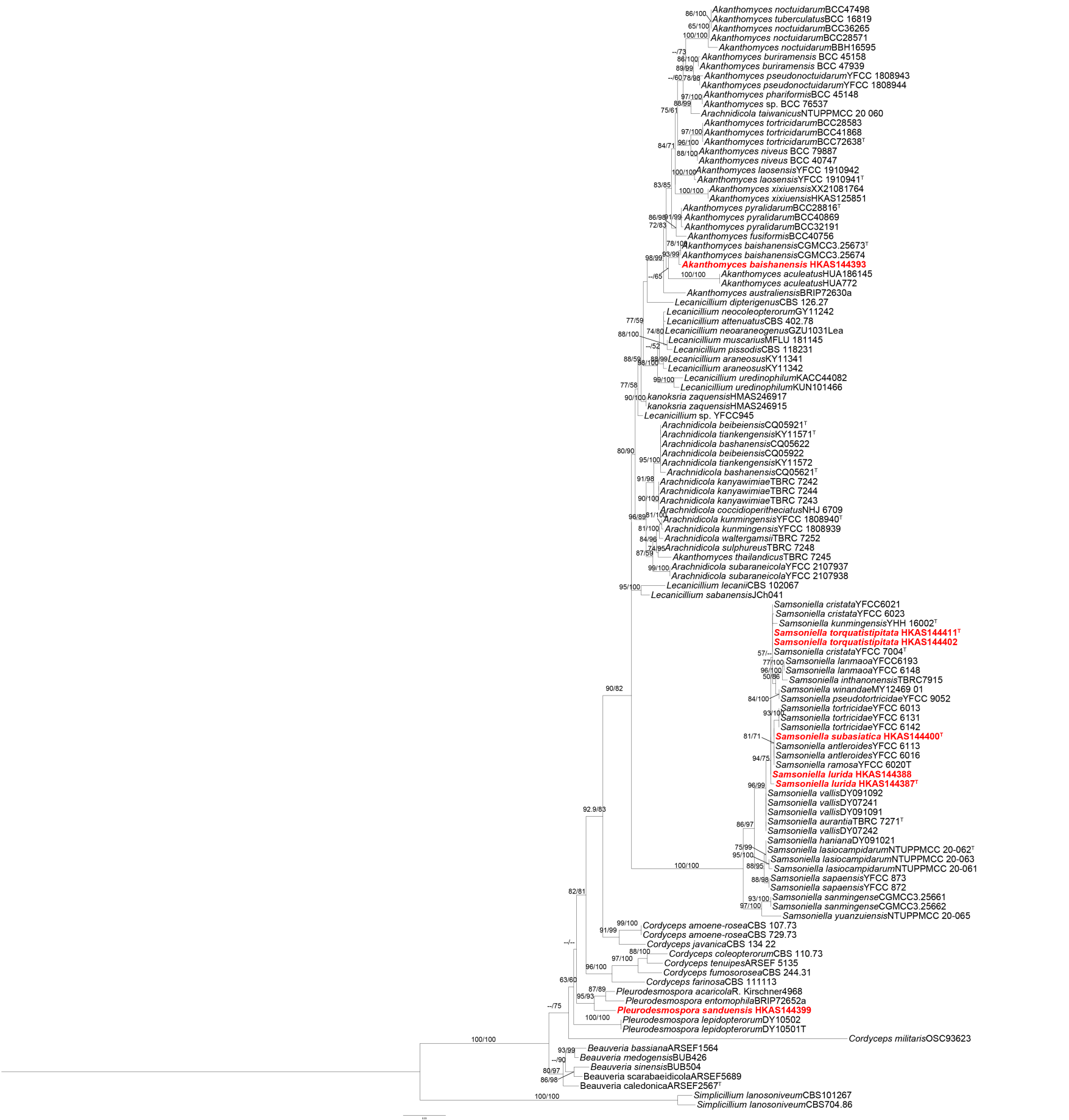


**Figure S4** The 3P_*TEF-*based tree in Fig. 1 of the manuscript. In this tree, each genus respectively forms monophyletic clade, supporting 3P_*TEF* is essential for investigation of generic relationship in this family. *Pleurodesmospora sanduensis* **sp. nov.** form a separated clade within pther species, supporting their novelty. However, insufficient genetic distances were observed for *Samsoniella* species. It is worth mentioning that *Cordyceps militaris* OSC 93623 (accession number: DQ522332) close to *Pleurodesmospora* as a independent clade instead of *Cordyceps*. This sequence maybe questionable.


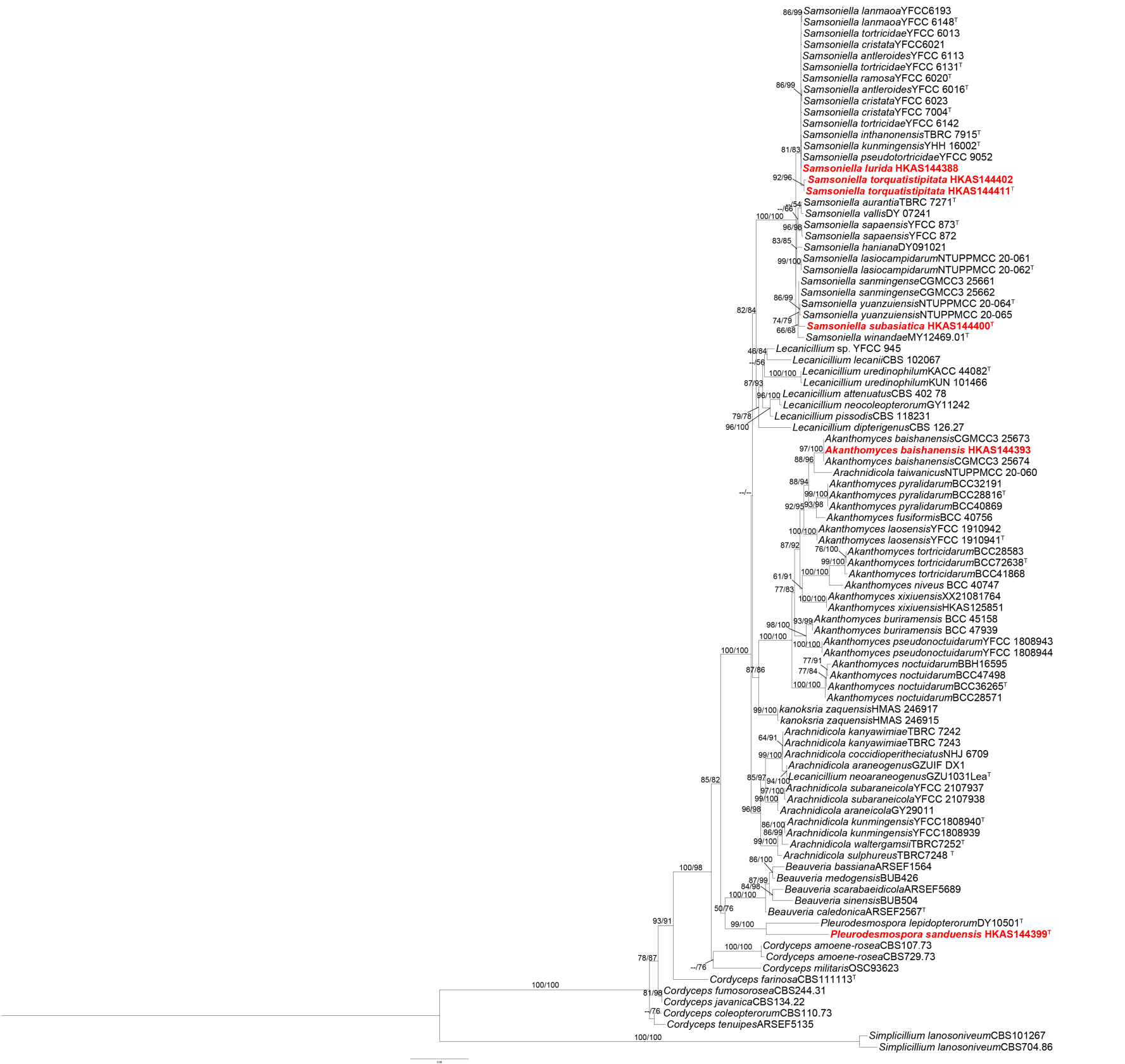


**Figure S5** The *rpb1*-based tree in Figure 1 of the manuscript. The topology inferred from *rpb1* is similar to that of 3P*_TEF*. *Pleurodesmospora sanduensis* **sp. nov.** (99% SH-aLRT / 100% UFB) is strongly supported as new species. However, phylogenetic relationships of *Samsoniella* species are ambiguous due to that all species of this genus cluster together without significant genetic distances.


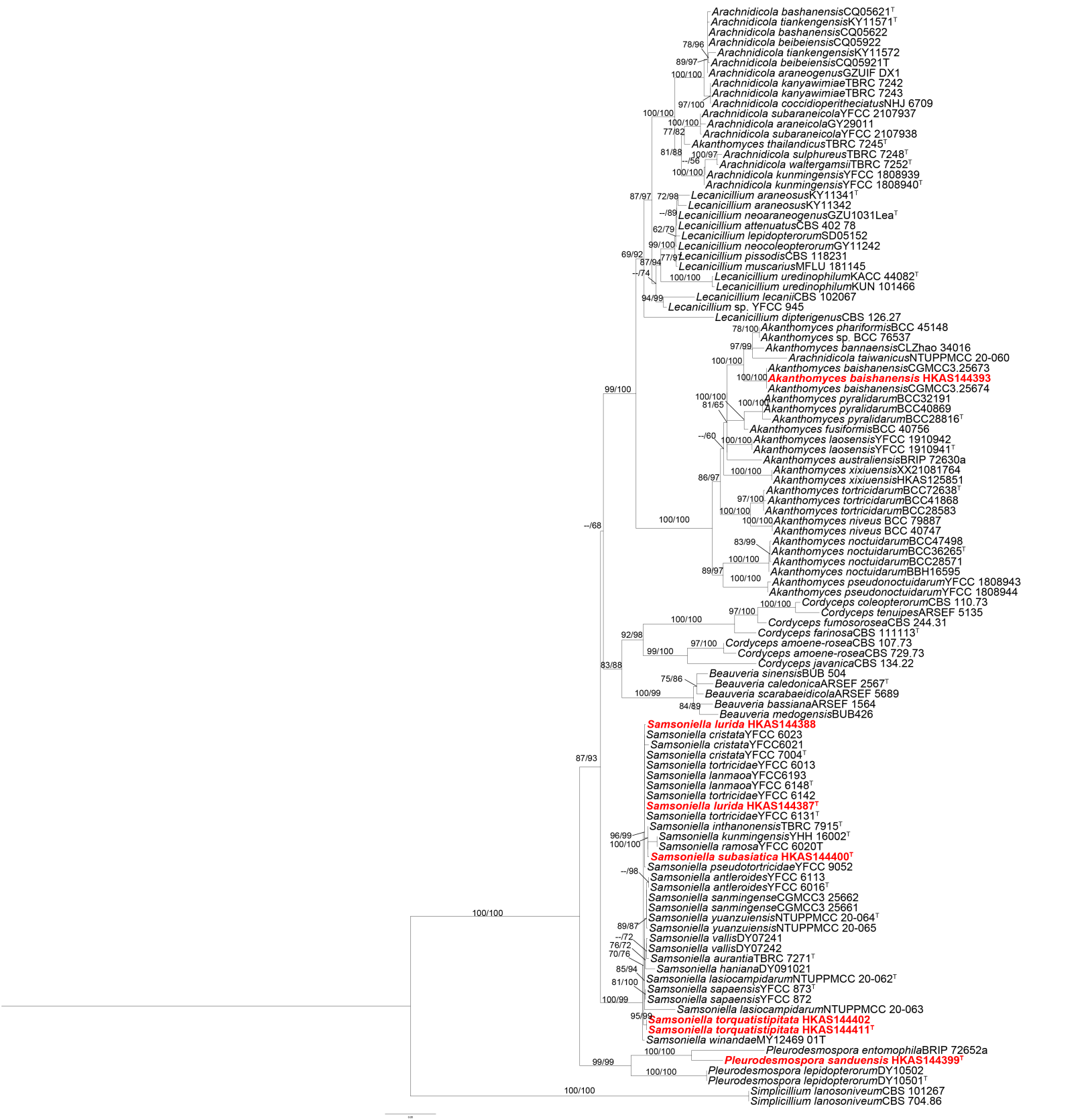


**Figure S6** The *rpb2*-based tree in Fig. 1 of the manuscript. Topology inferred from single *rpb2* is similar to those of 3P*_TEF* and *rpb1* gene. Each genus can be resolved as nonphylogenetic clade. *Pleurodesmospora sanduensis* **sp. nov.** (100% SH-aLRT / 100% UFB) is strongly supported as new species. However, phylogenetic relationships of *Samsoniella* species are ambiguous due to that all species of this genus cluster together without significant genetic distances.


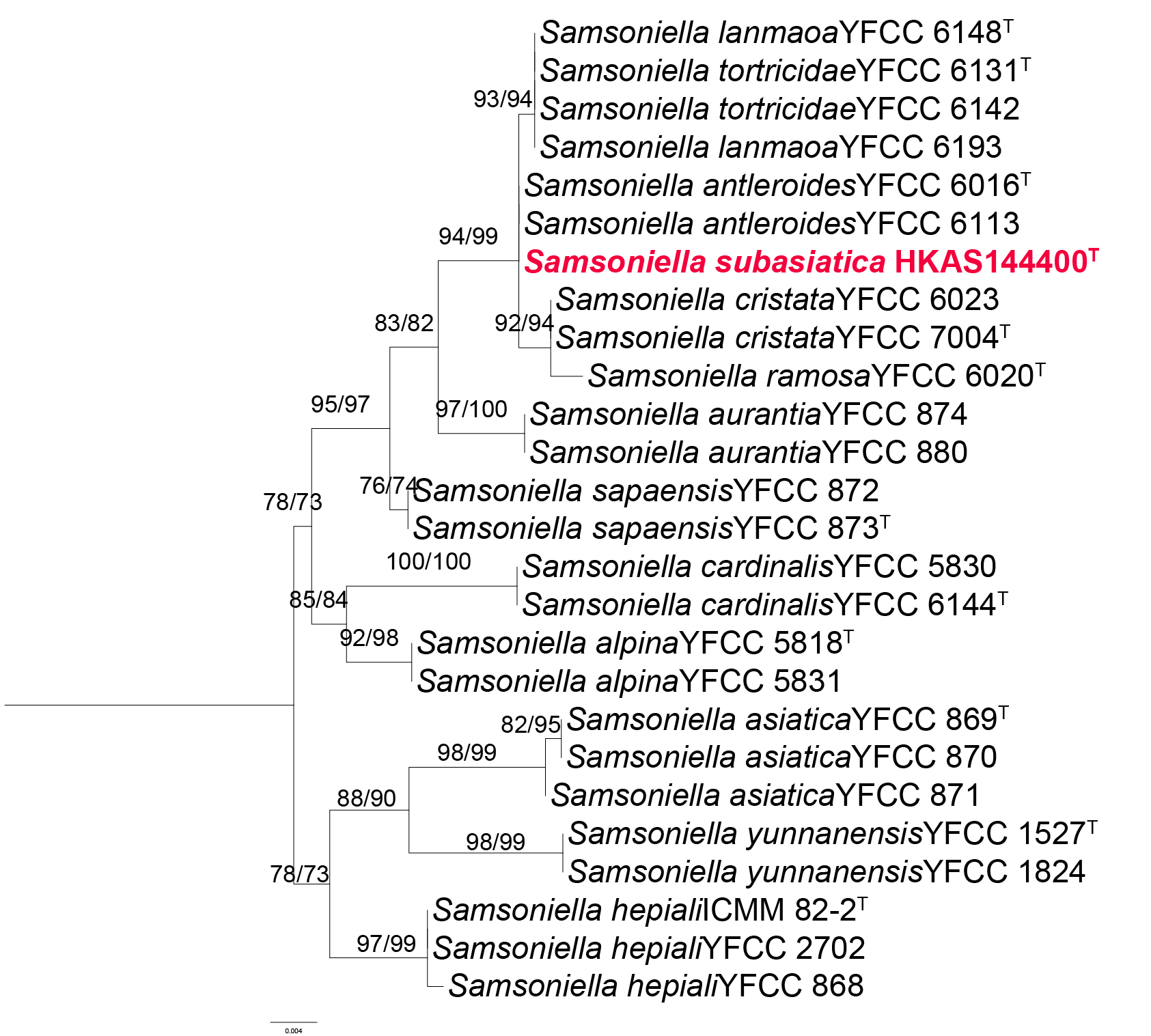


**Figure S7** The 5P*_TEF*-based tree in Fig. 2 of the manuscript. Among the five specimens of *Samsoniella* in this study, only HKAS144400 contains the 5P*_TEF* gene. *Samsoniella subasiatica* **sp. nov.** clusters with other *S. antleroides*. According to the results of the 5P*_TEF*-based tree, this gene can effectively distinguish some species within *Samsoniella*.


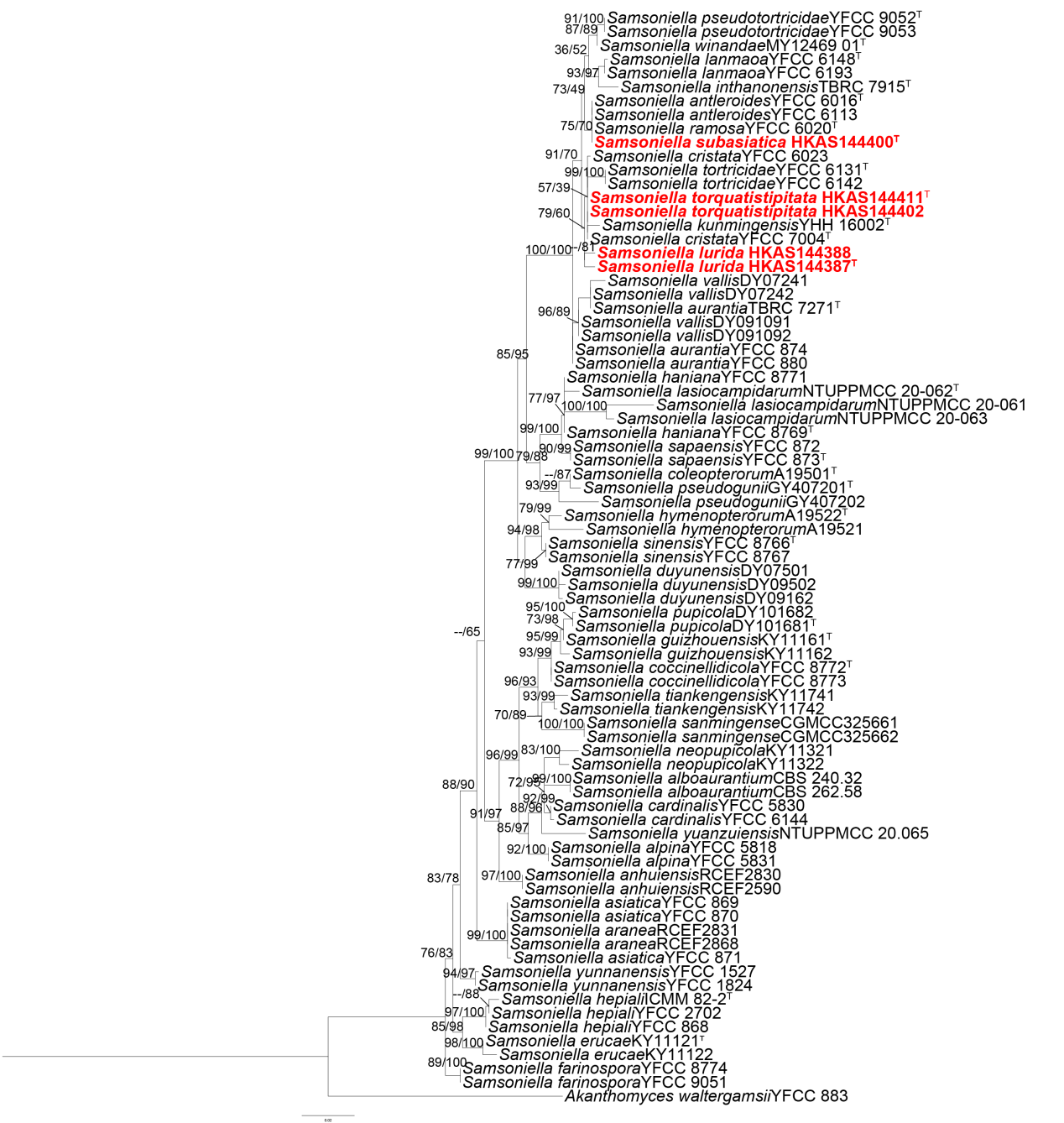


**Figure S8** The 3P*_TEF*-based tree in Fig. 2 of the manuscript. The new species *Samsoniella lurida* was placed in a distinct clade, supporting its novelty. However, *S. subasiatica* **sp. nov.** and *S. torquatistipitata* **sp. nov.** can not be separated from other species of *Samsoniella*.


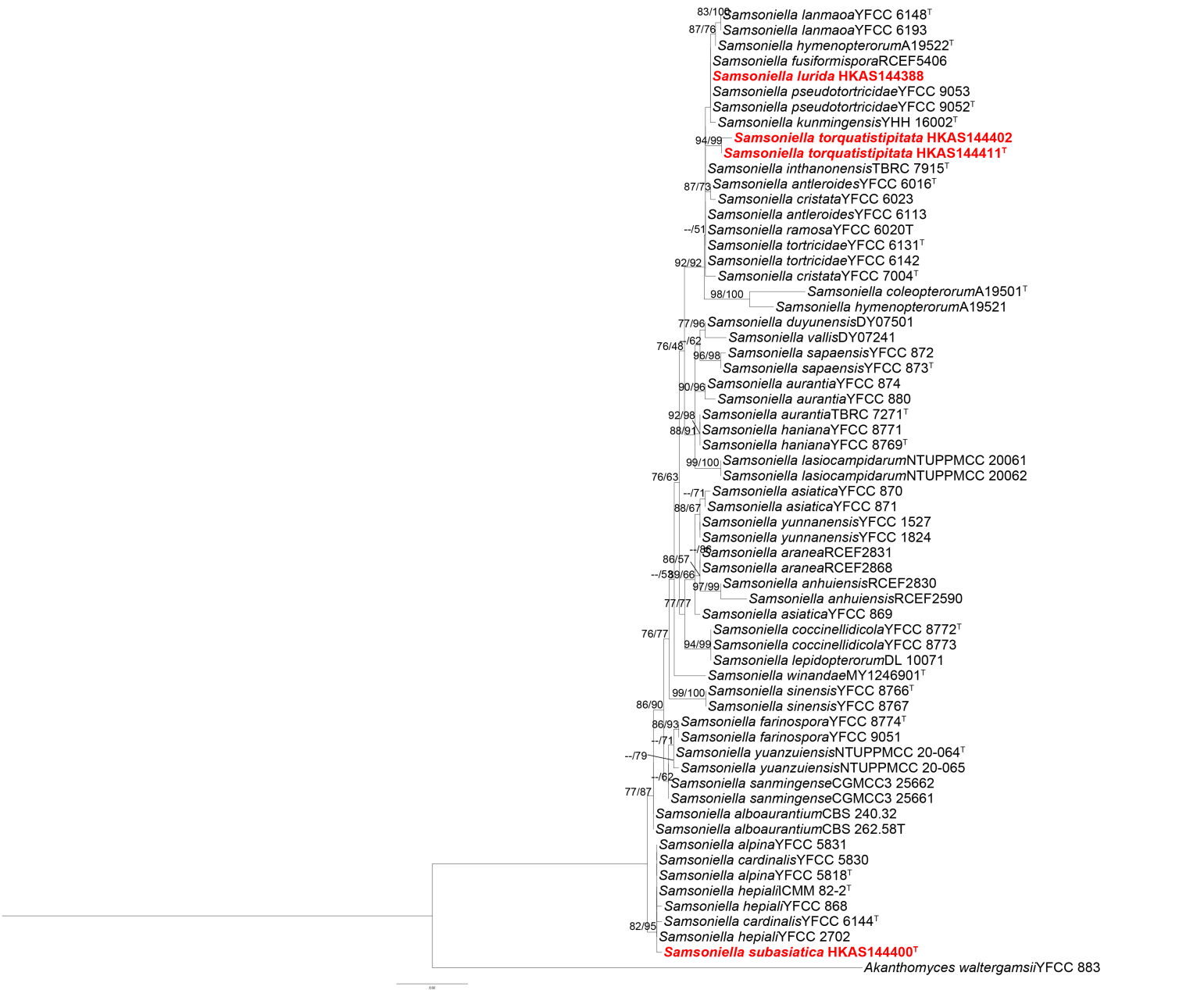


**Figure S9** The *rpb1*-based tree in Fig. 2 of the manuscript. *Samsoniella torquatistipitata* **sp. nov.** forms a separated clade with strong support (94% SH-aLRT / 99% UFB). However, *S. lurida* **sp. nov.** nor *S. subasiatica* HKAS 144400 **sp. nov.** can not be separated from other species of *Samsoniella*.


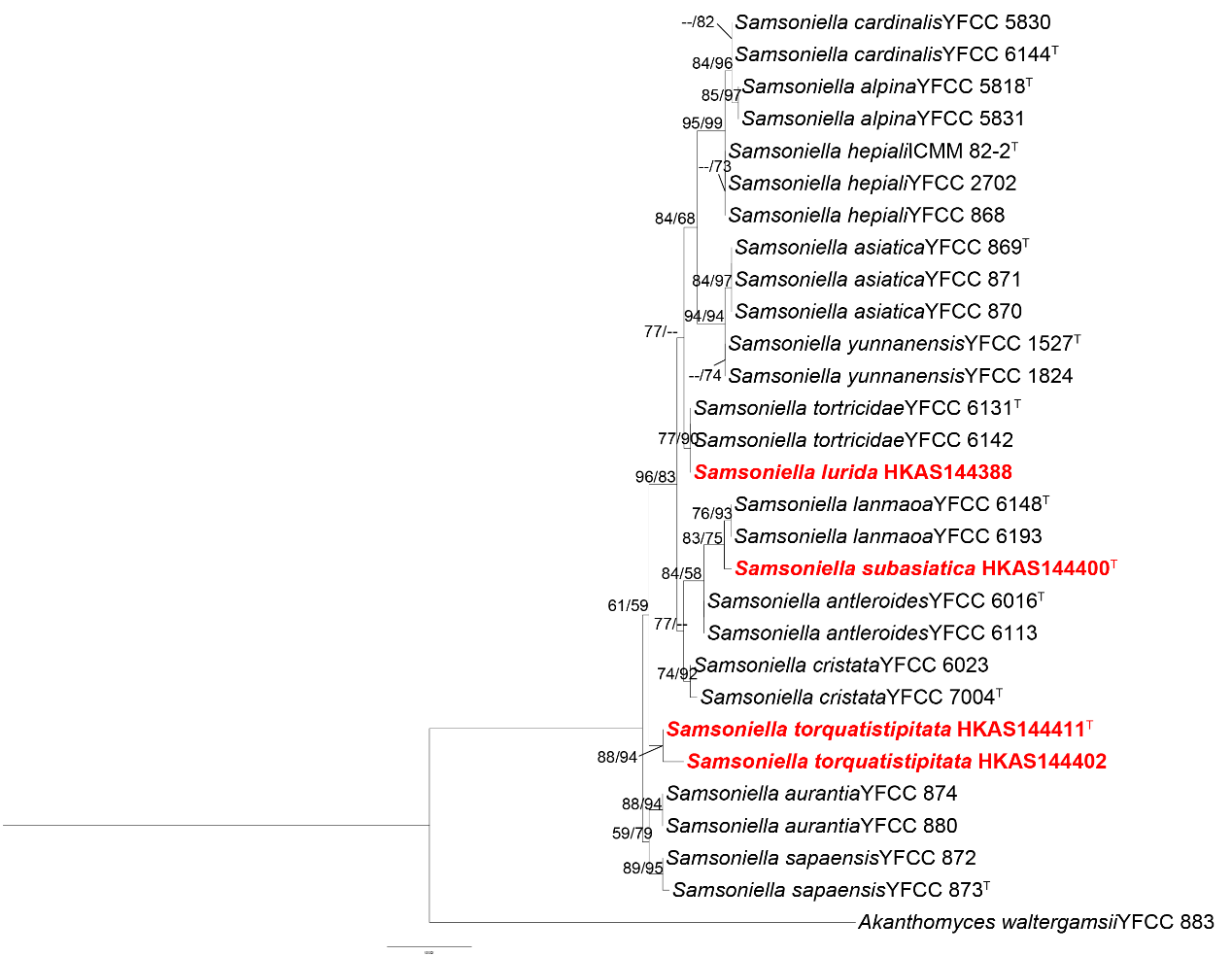


**Figure S10** The *MCM7*-based tree in Fig. 2 of the manuscript. *Samsoniella torquatistipitata* **sp. nov.** and *S. subasiatica* **sp. nov.** can be differentiated from other species with strong support (88% SH-aLRT / 94% UFB) and (83% SH-aLRT / 75% UFB), respectively. *Samsoniella lurida* **sp. nov.** clusters with other *S. tortricidae*.
